# Krill Oil Supplementation Reduces Exacerbated Hepatic Steatosis Induced by Thermoneutral Housing in Mice with Diet-Induced Obesity

**DOI:** 10.3390/nu13020437

**Published:** 2021-01-29

**Authors:** Gabriella Sistilli, Veronika Kalendova, Tomas Cajka, Illaria Irodenko, Kristina Bardova, Marina Oseeva, Petr Zacek, Petra Kroupova, Olga Horakova, Karoline Lackner, Amalia Gastaldelli, Ondrej Kuda, Jan Kopecky, Martin Rossmeisl

**Affiliations:** 1 Institute of Physiology of the Czech Academy of Sciences, Videnska 1083, 14220 Prague 4, Czech Republic; gabriella.sistilli@fgu.cas.cz (G.S.); veronika.kalendova@fgu.cas.cz (V.K.); tomas.cajka@fgu.cas.cz (T.C.); illaria.irodenko@fgu.cas.cz (I.I.); kristina.bardova@fgu.cas.cz (K.B.); marina.oseeva@fgu.cas.cz (M.O.); petra.kroupova@fgu.cas.cz (P.K.); olga.horakova@fgu.cas.cz (O.H.); ondrej.kuda@fgu.cas.cz (O.K.); jan.kopecky@fgu.cas.cz (J.K.); 2 Department of Physiology, Faculty of Science, Charles University, Vinicna 7, 12844 Prague 2, Czech Republic; 3 Proteomics Core Facility, Faculty of Science, Charles University, Division BIOCEV, Prumyslova 595, 25250 Vestec, Czech Republic; zacek@natur.cuni.cz; 4 Institute of Pathology, Medical University of Graz, Neue Stiftingtalstraße 6, 8010 Graz, Austria; karoline.lackner@medunigraz.at; 5 Cardiometabolic Risk Unit, Institute of Clinical Physiology, National Research Council, Via Moruzzi 1, 56100 Pisa, Italy; amalia@ifc.cnr.it

**Keywords:** NAFLD, obesity, omega-3, krill oil, phospholipids, high-fat diet, C57BL/6N mice, thermoneutral temperature

## Abstract

Preclinical evidence suggests that n-3 fatty acids EPA and DHA (Omega-3) supplemented as phospholipids (PLs) may be more effective than triacylglycerols (TAGs) in reducing hepatic steatosis. To further test the ability of Omega-3 PLs to alleviate liver steatosis, we used a model of exacerbated non-alcoholic fatty liver disease based on high-fat feeding at thermoneutral temperature. Male C57BL/6N mice were fed for 24 weeks a lard-based diet given either alone (LHF) or supplemented with Omega-3 (30 mg/g diet) as PLs (krill oil; ω3PL) or TAGs (Epax 3000TG concentrate; ω3TG), which had a similar total content of EPA and DHA and their ratio. Substantial levels of TAG accumulation (~250 mg/g) but relatively low inflammation/fibrosis levels were achieved in the livers of control LHF mice. Liver steatosis was reduced by >40% in the ω3PL but not ω3TG group, and plasma ALT levels were markedly reduced (by 68%) in ω3PL mice as well. Krill oil administration also improved hepatic insulin sensitivity, and its effects were associated with high plasma adiponectin levels (150% of LHF mice) along with superior bioavailability of EPA, increased content of alkaloids stachydrine and trigonelline, suppression of lipogenic gene expression, and decreased diacylglycerol levels in the liver. This study reveals that in addition to Omega-3 PLs, other constituents of krill oil, such as alkaloids, may contribute to its strong antisteatotic effects in the liver.

## 1. Introduction

Obesity is frequently associated with non-alcoholic fatty liver disease (NAFLD), a spectrum of conditions ranging from increased intrahepatic accumulation of triacylglycerols (TAGs; i.e., fatty liver or hepatic steatosis) to steatohepatitis (NASH) and end-stage liver disease [1]. Prevalence of hepatic steatosis and NASH in extremely obese subjects may reach up to 85% and 40%, respectively [2,3], while the presence of metabolic syndrome is associated with a potentially progressive, severe liver disease [4,5]. NAFLD is a serious public health problem [6] for which currently no approved drug therapy exists [7].

Dietary fatty acids (FAs) can differentially affect the body’s ability to store lipids in certain fat depots as well as in extra-adipose tissues [8]. In humans, overeating saturated FAs (SFAs) promoted hepatic and visceral fat storage [9,10,11]. Differential effects of various types of FAs are also observed in the case of regulation of inflammatory responses; thus SFAs and polyunsaturated FAs (PUFAs) of n-6 series are more pro-inflammatory, while PUFAs of n-3 series (omega-3 PUFAs) such as docosahexaenoic acid (DHA; 22:6n-3) and eicosapentaenoic acid (EPA; 20:5n-3) exert anti-inflammatory and hypolipidemic effects ([12,13,14], and reviewed in [15,16,17]). At the same time, omega-3 PUFA supplementation may reduce de novo lipogenesis (DNL) and increase FA oxidation in the liver [18], with the transcription factor peroxisome proliferator-activated receptor (PPAR)α playing a crucial role in the latter effect [19]. For these reasons, omega-3 PUFA supplements could be effective in preventing and treating NAFLD [20]. Indeed, in NAFLD patients treated with EPA and DHA as ethyl esters, a decrease in the percentage of liver fat was linearly correlated with the amount of omega-3 PUFAs taken [21]; however, no improvement in markers of liver function/injury or the fibrosis scores was detected. Similar results were obtained in subjects with non-cirrhotic NASH treated with omega-3 PUFAs as TAGs [22], in which a decrease in liver fat but no improvements in histological activity were observed. In general, omega-3 PUFAs administered in the form of TAGs or ethyl esters have been shown to partially limit hepatic steatosis in some studies [23].

Omega-3 PUFAs also alleviated hepatic steatosis in various rodent models of obesity (e.g., [14,24,25,26], and reviewed in [27]). Interestingly, the efficacy of omega-3 PUFAs may depend on the lipid form of their supplementation. For instance, compared to their TAG form, omega-3 PUFAs administered via phosphatidylcholine-rich phospholipids (PLs), either in the form of krill oil extracted from the Antarctic krill *Euphausia superba* [28] or as an extract of herring meal [26], had stronger effects in reducing the TAG content in the liver of rodents with genetically- or diet-induced obesity [25,26,29]. Moreover, in mice fed a corn oil-based high-fat diet, a significant reduction in hepatic TAGs was achieved only by administration of marine PLs containing omega-3 PUFAs and not soybean-derived phosphatidylcholine that did not contain EPA or DHA [30]. The higher efficacy of omega-3 PUFA-containing PLs in reducing hepatic steatosis could be related to the improved bioavailability of omega-3 PUFAs, in particular EPA and docosapentaenoic acid (22:5n-3), both in plasma and in target organs ([26,29,31] and reviewed in [32]). At the same time, supplementation of omega-3 PUFAs as PLs led to a stronger downregulation of liver gene expression in the DNL pathway [29,33,34] and significantly reduced activities of the corresponding lipogenic enzymes as well as of the mitochondrial citrate carrier [35]. However, despite its strong effects on TAG accumulation in the liver, it is not clear whether administration of omega-3 PUFAs in the form of PLs is able to affect advanced stages of NAFLD such as NASH and fibrosis, which remain unaffected in response to more traditional forms of omega-3 PUFAs such as TAGs or ethyl esters (see above).

Recently, a mouse model of obesity-associated exacerbated NAFLD based on the administration of a lard-based high-fat diet in a thermoneutral environment was introduced [36]. This experimental model is characterized by lower stress-driven production of corticosterone, augmented mouse pro-inflammatory immune responses and markedly exacerbated high-fat diet-induced NAFLD pathogenesis, which should recapitulate the severe end of the disease spectrum in humans. Thus, in the present study, the above experimental conditions were used to examine whether omega-3 PUFAs supplemented as PLs via krill oil could beneficially affect NAFLD-related phenotypes and hepatic insulin sensitivity, and what is the potential mechanism of action. At the same time, for comparison, other mice were administered omega-3 PUFAs in the form of a TAG-based concentrate, which was similar to krill oil in terms of the amount and ratio of EPA and DHA, thus representing the group receiving omega-3 PUFAs in one of the traditional lipid forms used for this purpose. 

## 2. Materials and Methods 

### 2.1. Animals and Diets

Male C57BL/6N mice (Charles River Laboratories, Sulzfeld, Germany) were obtained at the age of ~10 weeks. After arrival, mice were individually housed in cages and maintained at ~22 °C on a 12-h light/dark cycle (light from 6:00 a.m.), with ad libitum access to water and a standard diet (Chow; ~14 kJ/g; fat content ~3.6% (*w/w*); Rat/Mouse-Maintenance extrudate; ssniff Spezialdiäten GmbH, Soest, Germany). After one week of adaptation, the animals were transferred to a thermoneutral environment (~30 °C) and fed the following experimental diets: (i) a lard-based high-fat diet (LHF diet; ~21 kJ/g; fat content ~35% (*w/w*); product “DIO-60 kJ% fat (Lard)”, Cat. No. E15742-34; ssniff Spezialdiäten GmbH, Soest, Germany), (ii) a LHF-based diet supplemented with omega-3 PUFA-containing PLs (ω3PL diet), using krill oil (Rimfrost Sublime; EPA ~13%, DHA ~8%; Rimfrost AS, Ålesund, Norway), and (iii) a LHF-based diet supplemented with omega-3 PUFAs in the form of re-esterified TAGs (ω3TG diet), using the product Epax 3000 TG (EPA ~18%, DHA ~11%; Epax Norway AS, Ålesund, Norway). Experimental diets were prepared at the ssniff facility in Germany. The total content of EPA and DHA in both supplemented diets was ~30 mg/g diet. For details on macronutrient and FA composition of the experimental diets, see Supplementary Materials Tables S1 and S2, respectively. 

### 2.2. Experimental Setup

**Figure 1:**
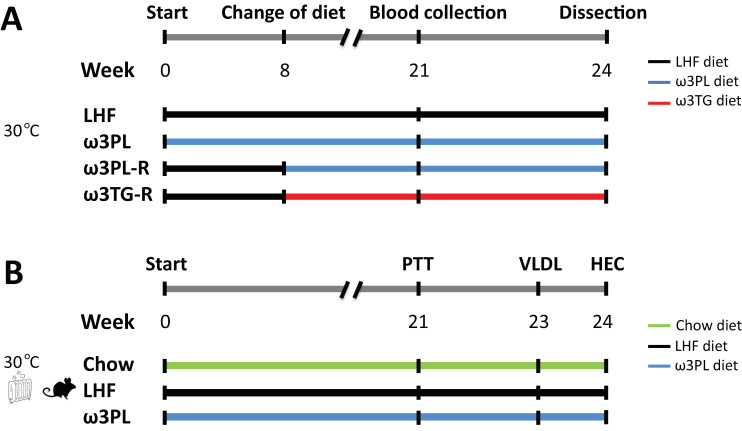
Overview of the experimental setup. (**A**) Four groups of mice (*n* = 8) housed in a thermoneutral environment (~30 °C) were used: (i) the control LHF group, which was fed a lard-based high-fat diet (i.e., LHF diet) for 24 weeks; (ii) ω3PL group fed a LHF-based diet supplemented with omega-3 PUFAs as PLs in the form of krill oil (i.e., ω3PL diet) for the duration of the experiment (i.e., “preventive” approach); (iii) ω3PL-R group fed the LHF diet for the first eight weeks and then from the ninth week on the ω3PL diet until the end of the experiment (i.e., “reverse” approach; marked with the letter “R” at the end of the group name); and (iv) ω3TG-R group fed the LHF diet for the first 8 weeks and then from the ninth week on the LHF-based diet supplemented with omega-3 PUFAs in the form of a concentrate of re-esterified TAGs (i.e., ω3TG diet) until the end of the experiment. (**B**) Three groups of mice (*n* = 8) housed in a thermoneutral environment (~30 °C) were used: (i) Chow group, in which mice were fed a standard low-fat diet and served as lean controls; (ii) the control LHF group, which was fed a lard-based high-fat diet (i.e., LHF diet) for 24 weeks; and (iii) ω3PL group fed the ω3PL diet for the duration of the experiment. Further details in Section 2.2. PTT, pyruvate tolerance test; VLDL, liver VLDL-TAGs secretion test; HEC, hyperinsulinemic-euglycemic clamp.

The experimental setup is shown in Figure 1. After moving to a thermoneutral environment, the mice were divided into four groups with the same average weight (*n* = 8; see Figure 1A). One group was fed the LHF diet for 24 weeks, as was the group fed the ω3PL diet from the beginning of the experiment (i.e., “preventive” approach). However, the other two groups first received the LHF diet for 8 weeks, and only then was this diet replaced with omega-3 PUFA-supplemented diets (ω3TG or ω3PL) administered for the remaining 16 weeks (i.e., “reverse” approach; marked with the letter “R” at the end of the group name). The total duration of all dietary interventions was therefore 24 weeks. Chow-fed mice served as lean controls. Body weight was recorded weekly and a fresh ration of diet was administered every other day. The calculation of cumulative energy intake was based on weekly measurements of food consumption over 24 h. Fasting plasma insulin and blood glucose levels were measured at week 21. Mice were killed by cervical dislocation under diethyl ether anesthesia between 9:00 a.m. and 11:00 a.m. Truncal blood was collected into tubes containing EDTA for plasma isolation, liver and white adipose tissue (WAT) samples from the epididymal, mesenteric and subcutaneous (dorso-lumbar) fat depots were dissected, weighed, and adiposity index was calculated as the sum of the weights of all analyzed WAT depots divided by body weight. Liver and epididymal WAT samples were snap-frozen in liquid nitrogen and stored at −80 °C for subsequent analyses, while one aliquot of tissue was used for histological evaluation. In a separate study using mice from the Chow, LHF and ω3PL groups (see Figure 1B), pyruvate tolerance, hepatic production of TAGs contained in very low-density lipoproteins (VLDL) and insulin sensitivity were determined at weeks 21, 23, and 24, respectively. Animal experiments were approved by the Institutional Animal Care and Use Committee and the Committee for Animal Protection of the Ministry of Agriculture of the Czech Republic (Approval Number: 81/2016).

### 2.3. Pyruvate Tolerance Test

The level of gluconeogenesis was estimated using pyruvate tolerance test. Mice fasted overnight (~14 h) were injected i.p. with pyruvate (1.5 mg/g body weight) and blood glucose levels were measured using glucometers Contour Plus (Bayer, Leverkusen, Germany) at time 0 (i.e., before injection), and then 15, 30, 60, 120, and 180 min after injection. The response to pyruvate administration was quantified as area under the glucose curve (AUC).

### 2.4. Light Microscopy and Immunohistochemical Analysis

Liver and epididymal WAT samples were fixed in 4% formaldehyde, embedded in paraffin, and sections of 5 µm thickness were stained using hematoxylin-eosin. The NAFLD histological scoring system [37] was used to assess the effect of omega-3 PUFAs administration on NAFLD progression. In epididymal WAT, macrophage marker MAC-2/galectin-3 was detected using specific antibodies (Cedarlane Laboratories; Burlington, NC, USA; 1:4000 dilution) and the number of crown-like structures (CLS) counted as before [38]. Morphometric analysis of WAT was performed using the imaging software NIS-Elements AR3.0 (Laboratory Imaging, Prague, Czech Republic).

### 2.5. Hepatic Production of VLDL-TAGs

The procedure was the same as before [39]. After an overnight fast (~16 h), mice were injected i.p. with a solution of 15% Tyloxapol (Triton WR-1339; Sigma-Aldrich; Prague, Czech Republic; dissolved in 0.9% saline) at a dose of 500 mg per kg body weight and blood was collected from the tail vein under basal conditions and 2, 4, and 6 h after Tyloxapol injection. Plasma TAG concentrations were measured at 500 nm using the Triglycerides kit from Erba Lachema (Brno, Czech Republic) and the Sunrise microplate reader (Tecan Group, Männedorf, Switzerland). 

### 2.6. Insulin Sensitivity Measured by Hyperinsulinemic-Euglycemic Clamp

Hyperinsulinemic-euglycemic clamp was performed in awake mice as before [14,29]. Briefly, a week before the end of the study, a permanent catheter was inserted into the *v. jugularis*. After a postoperative period of 4–7 days, mice were fasted for 6 h (6:00 a.m.–12:00 p.m.) and then infused with insulin Actrapid (Novo Nordisk) and D-[3-^3^H]glucose (Perkin Elmer, Boston, MA, USA) at a constant rate of 4.8 mU/min per kg body weight and 0.26 μCi/min, respectively. Euglycemia (~5.5 mmol/L) was maintained by periodical adjusting the variable infusion of glucose solution (30% for lean animals, 15% for obese animals), while blood glucose levels were regularly monitored using glucometers (see Section 2.3). Blood samples taken every 10 min during the last hour of the 3-h infusion period were used to analyze specific D-[3-^3^H]-glucose activity. 

### 2.7. Metabolites and Hormones

Plasma levels of lipid metabolites (i.e., TAGs, total cholesterol, non-esterified fatty acids), as well as aspartate transaminase (AST) and alanine aminotransferase (ALT), were measured using the appropriate assays from Roche or Wako (for the measurement of non-esterified fatty acids) and a Clinical Chemistry analyzer Roche/Hitachi 902 (Roche Diagnostics; Basel, Switzerland). Plasma levels of insulin were quantified using xMAP technology and MILLIPLEX MAP Mouse Metabolic Hormone Magnetic Bead Panel (MMHMAG-44K; Merck-Millipore; Burlington, MA, USA). Plasma levels of total adiponectin were measured by Mouse Adiponectin ELISA kit (EZMADP-60K; Sigma-Aldrich). Fasting plasma insulin and blood glucose levels were used to quantify Homeostatic Model Assessment of Insulin Resistance (HOMA-IR), using the following formula: fasting plasma insulin (mU/L) × fasting plasma glucose (mmol/L)/22.5.

### 2.8. TAG Content in the Liver 

Approximately 50 mg of tissue was dissolved in 150 µL 3M KOH (dissolved in 65% ethanol) at 70 °C for 2 h. The resulting homogenate was diluted 10× in redistilled water and the TAG content was measured (see Section 2.5) and the results were related to tissue weight.

### 2.9. Gene Expression Analysis

Gene expression was analyzed in the liver (stored in RNA later; Ambion, Austin, TX, USA) by real-time quantitative PCR as before [29,40]. Transcript levels were normalized to the expression level of a housekeeping gene for 18S ribosomal RNA (*Rn18s*). Gene names and sequences of the oligonucleotide primers are listed in Table S3.

### 2.10. Composition of FAs in Experimental Diets and Liver

Total lipids were extracted from aliquots of experimental diets (100 mg) by two-step extraction using hexane and a mixture of methanol and dichloromethane (see Supplementary Materials for details). The methyl tert-butyl ether (MTBE)-based extraction of total lipids from the liver (50 mg) was performed as before [31], and neutral and polar lipid fractions were obtained by using SPE Columns (Discovery). Trans-esterification of extracted lipids, FAs methyl esters (FAME) extraction and their analysis using comprehensive two-dimensional gas chromatography with mass detection (Pegasus 4D, LECO, USA) was performed as before [41].

### 2.11. LC-MS Analysis of Liver Samples

Metabolomic and lipidomic profiling of liver samples was conducted using a combined targeted and untargeted workflow for the lipidome, metabolome, and exposome analysis (LIMeX) [42,43]. Extraction was carried out using a biphasic solvent system of cold methanol, MTBE, and 10% methanol. Four different LC-MS platforms were used for metabolomic and lipidomic profiling: (i) lipidomics of complex lipids in positive ion mode, (ii) lipidomics of complex lipids in negative ion mode, (iii) metabolomics of polar metabolites in positive ion mode, and (iv) metabolomics of polar metabolites in negative ion mode. Details of sample preparation, LC-MS conditions, raw data processing and curation, and list of annotated complex lipids and polar metabolites are in Supplementary Materials.

### 2.12. Data Processing and Statistics

Results are means ± SEM. To compare the groups fed experimental LHF-based diets, One Way ANOVA (for normally distributed data sets) or Kruskal–Wallis test (non-normally distributed data sets) followed by Student–Newman–Keuls post-hoc test was used (SigmaStat 3.5 software; Systat Software Inc., San Jose, CA, USA). Differences were considered significant when *p* ≤ 0.05. Pearson’s correlation coefficient (*r*) was calculated to measure the strength of the association between the two variables. Multivariate analysis was performed using partial least-squares discriminant analysis (PLS-DA) using MetaboAnalyst 4.0 [44]. Statistical models were created for metabolomic and lipidomic sets separately after logarithmic transformation (base 10) and Pareto scaling. Exported variable importance in projection (VIP) scores were used for evaluation.

The number of animals required to evaluate the effect of krill oil supplementation on liver steatosis was based on a previous publication [29], namely the difference in liver TAGs between control mice fed a corn oil-based high-fat diet (i.e., cHF diet) and mice fed a cHF-based diet containing krill oil (i.e., the ω3PL-H diet), supplemented at the same dose as in the current study. Thus, the minimal sample size of 6 animals per group was calculated using G*Power software (power 0.95, α = 0.05; see [45]).

## 3. Results

### 3.1. Basic Parameters of Energy Balance, Adiposity, as well as Lipid and Glucose Homeostasis

**Figure 2:**
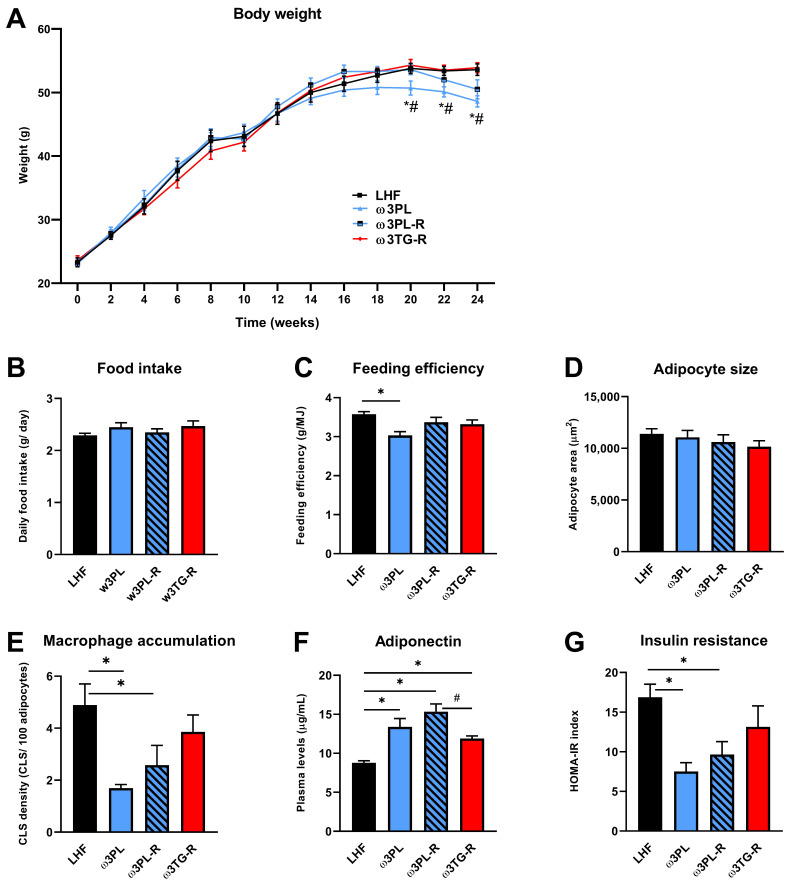
The effect of omega-3 PUFA supplementation on parameters related to energy balance, adipose tissue health and insulin sensitivity: changes in body weight during the study (**A**), average daily food intake (**B**), feeding efficiency (**C**), average size of adipocytes (**D**) and macrophage accumulation in epididymal WAT (**E**), plasma adiponectin levels (**F**), and insulin resistance based on the HOMA-IR index (**G**). Data are means ± SEM (*n* = 7–8). *, significant effect of omega-3 PUFAs (vs. LHF); #, significant difference from ω3TG-R (One Way ANOVA or Kruskal–Wallis).

Table 1 and Figure 2 show the general characteristics of obese mice from the control group (LHF) and the three intervention groups (ω3PL, ω3PL-R and ω3TG-R; description of groups—see Section 2.2 and Figure 1) that differed in the lipid form and/or timing of omega-3 PUFA administration. In the control LHF group, high-fat feeding for 24 weeks caused a weight gain of ~30 g, which was reduced by 15% in the ω3PL group supplemented with krill oil since the start of the study (Table 1); this reduction in weight gain was mainly due to weight loss during the second half of the study (Figure 2A). Although no changes in average daily food intake (Figure 2B) and cumulative energy intake (Table 1) were observed between the groups, the average feeding efficiency, calculated by dividing the weight gain by the amount of energy consumed each week, was significantly reduced in ω3PL mice (Figure 2C). The adiposity index did not change in response to omega-3 PUFA administration (Table 1), but specifically in the ω3PL group, mesenteric WAT weight was reduced by ~27% (a similar trend was also observed in ω3PL-R mice), while the weight of epididymal WAT was increased in the ω3PL and ω3PL-R groups (Table 1). Despite the increased weight of epididymal WAT in both krill oil-supplemented groups, the average size of adipocytes in this fat depot remained unchanged (Figure 2D) while tissue accumulation of inflammatory macrophages, analyzed by MAC-2/galectin-3 immunodetection (see Supplementary Figure S1) and assessed by CLS counting, was reduced (Figure 2E); no such changes were observed in the ω3TG-R group. With regard to lipid metabolism markers in the circulation, omega-3 PUFAs reduced total cholesterol levels, regardless of the lipid form of their supplementation (Table 1). Given the role of obesity and WAT inflammation in impaired glucose metabolism, we next evaluated the effect of omega-3 PUFAs on glucose homeostasis; it was improved specifically in krill oil-supplemented mice (i.e., ω3PL and ω3PL-R), as evidenced by lower FBG and non-fasting plasma insulin (Table 1). Furthermore, these mice also showed stronger induction of plasma adiponectin levels (Figure 2F) in association with significantly reduced HOMA-IR (Figure 2G). 

Thus, in obese mice kept in a thermoneutral environment, administration of krill oil, unlike omega-3 PUFAs supplemented in the TAG form, led to redistribution of WAT and improvement of its function, which corresponded to positive effects on glucose homeostasis. 

### 3.2. Histological Analysis of NAFLD-Related Phenotypes

**Figure 3:**
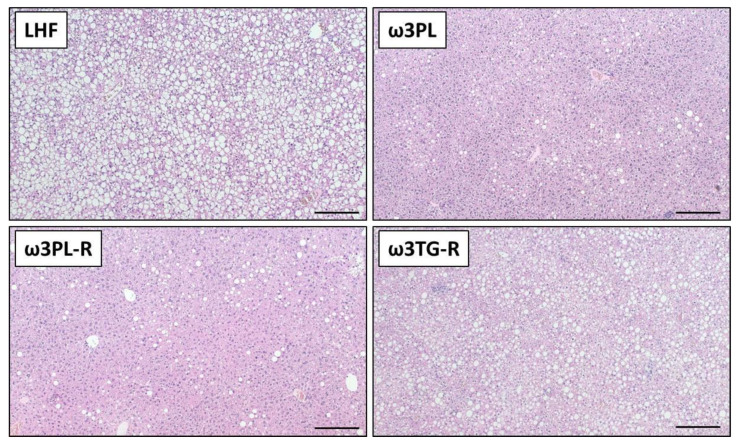
Representative histological sections of liver stained with hematoxylin and eosin. Bars = 200 µm.

**Figure 4:**
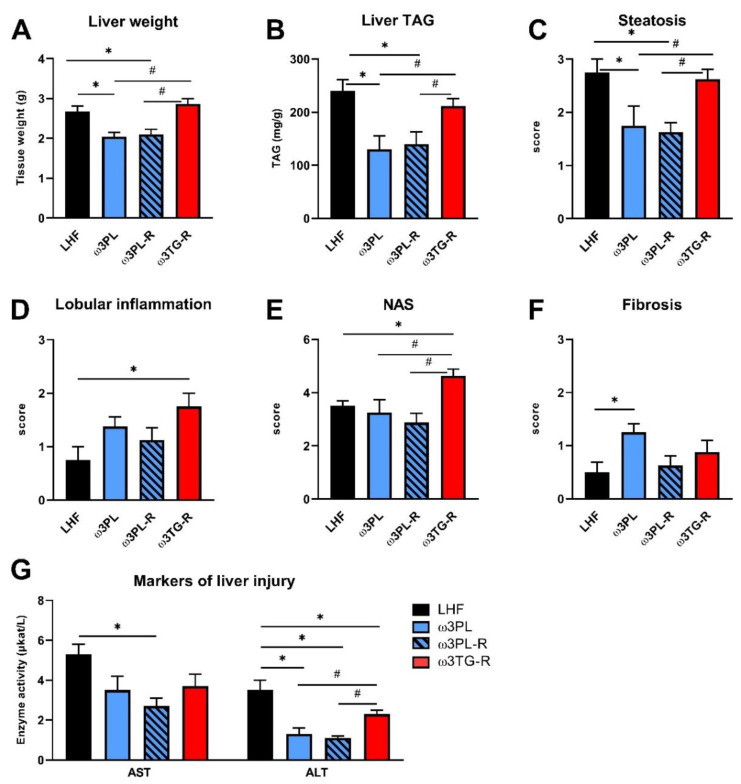
The effect of omega-3 PUFA supplementation on NAFLD-related parameters: liver weight (**A**), liver TAG content (**B**), steatosis (**C**), lobular inflammation (**D**), NAFLD activity score—NAS (**E**), fibrosis (**F**), and plasma AST and ALT levels (**G**). The results presented in panels C–F are based on histological analysis of liver sections. Data are means ± SEM (*n* = 7–8). *, significant effect of omega-3 PUFAs (vs. LHF); #, significant difference from ω3TG-R (one-way ANOVA or Kruskal–Wallis).

We further examined the effect of krill oil and omega-3 PUFAs supplemented as TAGs on the development of NAFLD using biochemical and histological analyses of the liver (Figure 3 and Figure 4). 

Despite having similar plasma TAG levels (Table 1), liver weight (Figure 4A) and the TAG content in the liver (Figure 4B; quantified biochemically) were reduced in both experimental groups that received krill oil compared either to LHF controls or ω3TG-R mice supplemented with omega-3 PUFAs as TAGs. Histological analysis of hematoxylin-eosin-stained liver sections (Figure 3) confirmed reduced levels of steatosis in the livers of ω3PL and ω3PL-R mice compared to the LHF and ω3TG-R groups, where the steatosis score reached almost the maximum value of 3 (Figure 4C). In addition to the degree of steatosis, histological analysis was also used to assess other components of the NAFLD activity score (NAS), which includes lobular inflammation and hepatocyte ballooning. Lobular inflammation was increased in ω3TG-R mice and unchanged in krill oil-supplemented mice compared to LHF-fed controls (Figure 4D), while hepatocyte ballooning was relatively less frequent (score < 0.5) and was similar among the groups (not shown). As a result, the NAS score was higher in ω3TG-R mice and unchanged in ω3PL and ω3PL-R animals compared to LHF-fed controls (Figure 4E). We also evaluated the degree of fibrotic changes in the liver, which was generally relatively low in all groups (Figure 4F); however, ω3PL mice supplemented with krill oil during the development of obesity showed significantly higher fibrosis score compared to control animals fed LHF (Figure 4F). On the other hand, plasma levels of transaminases, especially ALT, were reduced due to krill oil supplementation, with a weaker effect observed in mice receiving omega-3 PUFAs as TAGs (Figure 4G).

These data primarily document the excellent efficacy of krill oil in reducing severe hepatic steatosis induced by administration of a high-fat diet in a thermoneutral environment. 

### 3.3. Analysis of Parameters Related to the Efficacy of Omega-3 PUFAs in the Liver

**Figure 5:**
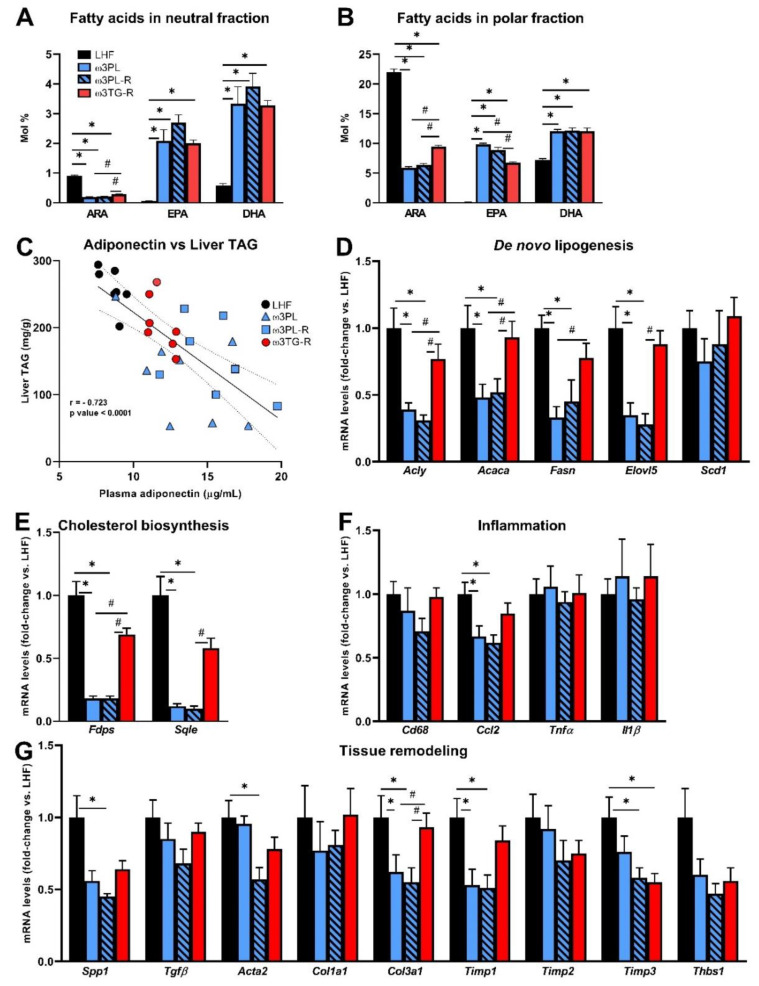
Selected parameters determining the effects of omega-3 PUFAs in the liver: bioavailability of FAs such as arachidonic acid (ARA), eicosapentaenoic acid (EPA) and docosahexaenoic acid (DHA) measured in the neutral (**A**) and polar (**B**) fraction of liver lipids; correlation between plasma adiponectin levels and the degree of TAG accumulation in the liver (**C**); hepatic expression of genes related to DNL (**D**), cholesterol biosynthesis (**E**), inflammation (**F**), and tissue remodeling (**G**). Data are means ± SEM (*n* = 7–8). *, significant effect of omega-3 PUFAs (vs. LHF); #, significant difference from ω3TG-R (one-way ANOVA or Kruskal–Wallis).

Next, we analyzed some parameters that may determine the metabolic effects of krill oil in the liver (Figure 5). First, the bioavailability of FAs such as arachidonic acid (ARA), EPA, and DHA, which are substrates for the formation of biologically active lipid mediators, was measured in the neutral (i.e., mainly TAGs) and polar (i.e., mainly PLs) lipid fractions of the liver (Figure 5A,B; for complete data on FAs composition in hepatic TAGs and PLs, see Tables S4 and S5, respectively). 

In general, the relative content of ARA was reduced while the content of EPA and DHA was increased in response to administration of both krill oil and omega-3 PUFAs as TAGs; however, the ARA reduction efficacy in both the neutral and polar fractions was significantly higher in the krill oil-supplemented groups (Figure 5A,B), and the same situation was observed in the case of an increase in EPA primarily in the polar fraction (Figure 5B). We also examined the relationship between plasma adiponectin levels and the degree of TAG accumulation in the liver (Figure 5C), as adiponectin can activate 5′-AMP-activated protein kinase (AMPK) and PPARα and thus stimulate lipid catabolism. Indeed, in the livers of mice fed various LHF-based diets, plasma adiponectin levels and liver TAG levels showed a strong negative correlation (*r* = −0.723; *p* < 0.0001; Figure 5C). 

Furthermore, the above findings regarding NAFLD-related phenotypes were then related to changes in gene expression of key enzymes of lipid and cholesterol metabolism, as well as markers of inflammation and tissue remodeling with a known relationship to the development of NAFLD/NASH (Figure 5D–G). Expression of the genes related to DNL (Figure 5D) and cholesterol biosynthesis (Figure 5E) was reduced up to 10-fold in krill oil-supplemented mice (ω3PL and ω3PL-R mice) compared to LHF-fed controls. In contrast, no such changes were observed in ω3TG-R mice receiving omega-3 PUFAs as TAGs (Figure 5D,E). Although histological analysis did not reveal a significant degree of NASH/fibrosis induced in C57BL/6N mice fed the LHF diet in a thermoneutral environment (Figure 4D,F), the expression of both inflammatory genes and tissue remodeling genes in the liver was analyzed in order to see whether supplementation with krill oil or omega-3 PUFAs as TAGs could affect these processes at this level. There were no significant differences between groups in the expression of inflammation-related genes (Figure 5F), with the exception of chemokine (C-C motif) ligand 2 (CCL2; also known as monocyte chemoattractant protein 1), whose expression was reduced in groups ω3PL and ω3PL-R. No consistent effects on the expression of genes related to tissue remodeling were observed (Figure 5G); in general, the administration of krill oil reduced the mRNA levels of some of the measured genes (e.g., Spp1, Col3a1, Timp1, Timp3), with occasional effects (e.g., Timp3) also observed in ω3TG-R mice given omega-3 PUFAs as TAGs. Decreased expression of genes involved in DNL may therefore help explain the beneficial effects of krill oil supplementation on liver fat accumulation, while its effect on the expression of inflammatory genes, and in particular tissue remodeling genes, was not conclusive.

### 3.4. In Vivo Analyses Related to Liver Function and Insulin Sensitivity

**Figure 6:**
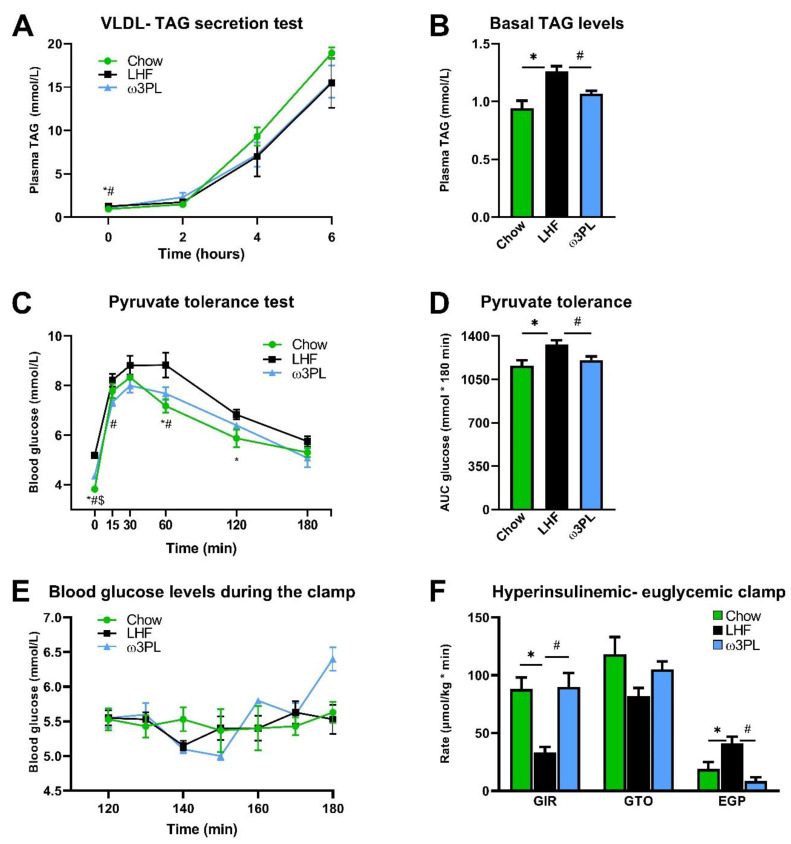
Effect of krill oil supplementation on hepatic VLDL-TAG production (**A**), fasting plasma TAG levels (**B**), glycemia during the pyruvate tolerance test (**C**), the level of pyruvate-driven gluconeogenesis (**D**), as well as glycemia during the last hour of hyperinsulinemic-euglycemic clamp (**E**) and clamp-related parameters including glucose infusion rate (GIR), glucose turnover (GTO) and endogenous glucose production (EGP; **F**). Data are means ± SEM (*n* = 6–7). *, significant difference between LHF and Chow; #, significant difference between LHF and ω3PL; $, significant difference between ω3PL and Chow (one-way ANOVA or Kruskal–Wallis).

Given the significant reduction in TAG levels in the livers of mice supplemented with krill oil (Figure 4B,C) and a likely reduction in insulin resistance (i.e., HOMA-IR; Figure 2G) in these animals, we initiated an additional experiment (see also Figure 1B), in which we performed a series of in vivo functional assays in mice fed either the Chow, LHF or ω3PL diet for 24 weeks at 30 °C (Figure 6). At the end of the study, mice in the Chow, LHF and ω3PL groups weighed 41.3 ± 1.1, 57.6 ± 0.5, and 54.5 ± 0.8 g (*p* < 0.01 vs. LHF for both other groups), which corresponded to a weight gain of 14.5 ± 1.0, 30.7 ± 0.4, and 27.5 ± 0.6 g (*p* < 0.0001 vs. LHF), respectively. Thus, the weight gain in LHF and ω3PL mice was similar to that observed in the previous experiment (see Table 1). We first evaluated whether lower levels of hepatic steatosis in ω3PL mice, supplemented with krill oil during the development of obesity, can be explained by changes in hepatic production of TAGs contained in VLDL (Figure 6A,B). However, the VLDL-TAG secretion test in overnight fasted mice did not reveal any significant differences in lipemic curves between the groups, especially LHF and ω3PL mice (Figure 6A), although basal plasma TAG levels were decreased by 15% in ω3PL compared to LHF mice (Figure 6B). We further examined whether the potent effect of krill oil supplementation on hepatic steatosis is associated with changes in gluconeogenesis and/or improved insulin sensitivity. Thus, we evaluated the glycemic response to pyruvate injection to determine gluconeogenic activity; glycemic curves (Figure 6C), as well as quantification of the glycemic response based on AUC values (Figure 6D), indicated a reduced level of pyruvate-stimulated gluconeogenesis in both Chow and ω3PL mice compared to LHF-fed controls. Finally, we used the state-of-the-art hyperinsulinemic-euglycemic clamp technique in combination with a radioactive glucose tracer to analyze whole-body and hepatic insulin sensitivity (Figure 6E,F). Whole-body insulin sensitivity was markedly impaired in obese LHF-fed control animals compared to Chow-fed mice, as documented by changes in glucose infusion rate (GIR; Figure 6F), reflecting the amount of exogenous glucose required to maintain euglycemia during the clamp (i.e., under insulin-stimulated conditions) and which showed a ~2.7-fold reduction in LHF mice. 

However, the total glucose turnover (GTO) in the organism was not significantly changed in LHF mice compared to the Chow group, primarily because of an ~2.2-fold increase in endogenous glucose production (EGP; Figure 6F). In contrast, krill oil supplementation in ω3PL mice led to normalization of insulin sensitivity at the whole-body level and in the liver, as shown by increased GIR and decreased EGP levels under hyperinsulinemic conditions (Figure 6F). Therefore, the above data suggest that the potent antisteatotic effects of krill oil supplementation in the livers of mice with diet-induced obesity and exacerbated hepatic steatosis are associated with improved whole-body and tissue sensitivity to insulin, but cannot be explained by changes in VLDL-TAG secretion. 

### 3.5. Hepatic Metabolome in Relation to Tissue TAG Accumulation and Insulin Sensitivity

**Figure 7:**
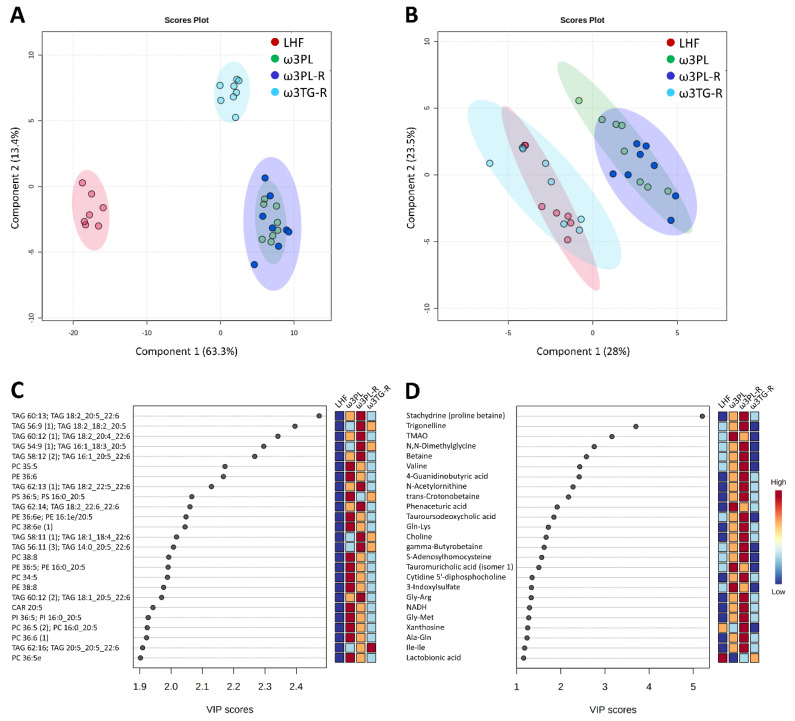
Four-class PLS-DA score plots of complex lipids (**A**; *n* = 507) and polar metabolites (**B**; *n* = 157) in the liver in response to dietary challenges, and the most discriminating complex lipids (**C**) and polar metabolites (**D**) based on VIP scores from PLS-DA.

To better understand the underlying mechanisms of the potent antisteatotic and insulin-sensitizing effects of krill oil supplementation in mice with exacerbated hepatic steatosis, and to determine how these mechanisms differ in mice given omega-3 PUFAs as TAGs (see also Figure 1A), we analyzed the metabolipidomic profiles of the liver using four different LC-MS platforms (see Section 2.11 for details). First, the annotated data were analyzed using PLS-DA, supervised classification technique, to gain a general view on the impact of omega-3 PUFAs supplementation on both complex lipids and polar metabolites (Figure 7; see Table S7 for a complete list of annotated metabolites). 

**Figure 8:**
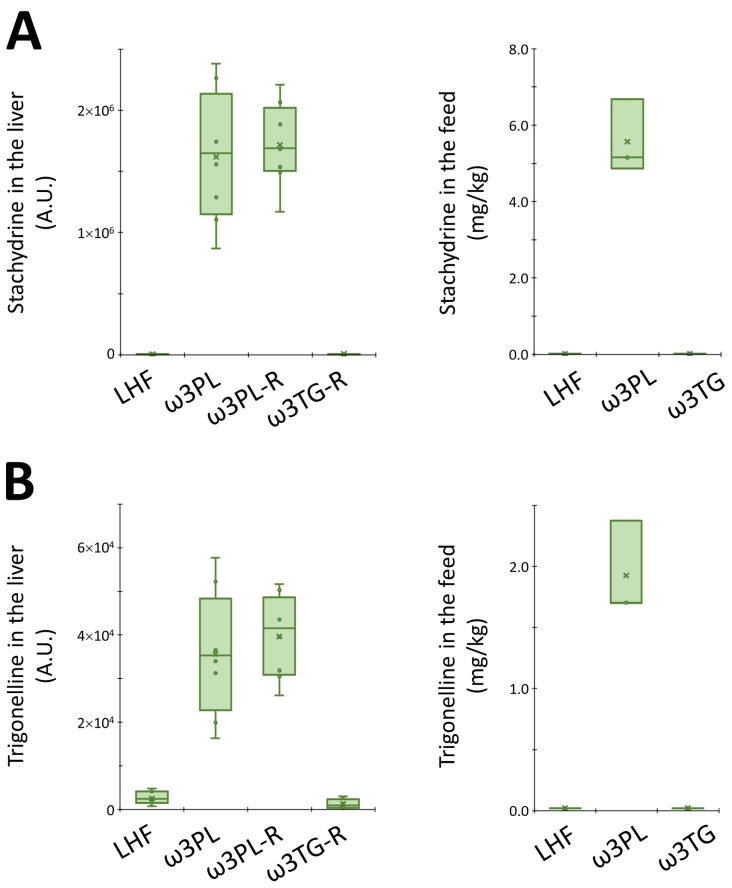
Box plots for stachydrine (**A**) and trigonelline (**B**) levels in the liver (arbitrary units; left panel) and in experimental diets (mg/kg; right panel).

In the case of complex lipids (Figure 7A), the analysis revealed a distinct separation of both krill oil-supplemented mice and mice given omega-3 PUFAs as TAGs from LHF-fed controls within the first Component (C1), which describes 63% of the total variation between these groups. Moreover, both groups fed with krill oil (i.e., ω3PL, ω3PL-R) separated from the ω3TG-R group (omega-3 PUFAs as TAGs) within C2 (Figure 7A). A separate analysis of polar metabolites (Figure 7B) showed a weaker separation of mice fed with krill oil from both LHF controls and mice fed a diet containing omega-3 PUFAs as TAGs within C1, which accounted for 28% of the total variation between the LHF and ω3TG-R groups on the one hand and the ω3PL and ω3PL-R groups on the other. Thus, krill oil supplementation had major effects on both complex lipids and polar metabolites, with omega-3 TAGs affecting mainly the lipidome. Subsequent VIP analysis indicated that several TAG species containing DHA and/or EPA (e.g., TAG 60:13; TAG 18:2_20:5_22:6, TAG 56:9; TAG 18:2_18:2_20:5 or TAG 60:12; TAG 18:2_20:4_22:6) were the most discriminating factors in terms of complex lipids (Figure 7C), while alkaloids stachydrine and trigonelline, as well as trimethylamine N-oxide (TMAO), represented the most discriminating factors among polar metabolites (Figure 7D). In the case of alkaloids, their concentration was significantly increased in the liver of ω3PL and ω3PL-R mice compared to the LHF and ω3TG mice (see left panels in Figure 8A,B), which is due to the increased concentration of these substances in the krill oil-containing diet (right panels in Figure 8A,B).

**Figure 9:**
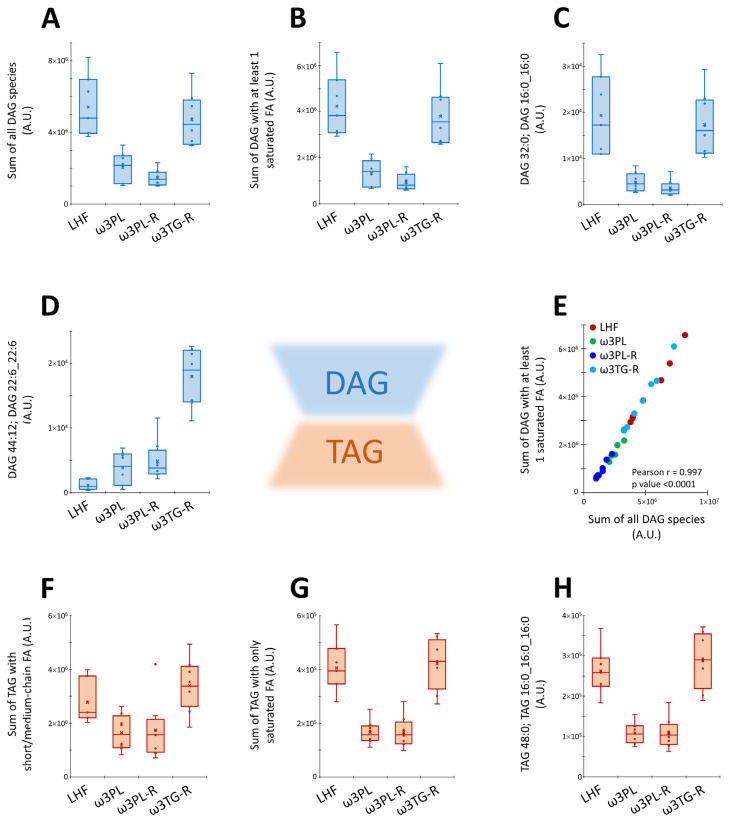
Box plots and correlation analysis for DAGs and TAGs lipid classes in the liver in response to dietary challenges: the sum of all DAG species (**A**; *n* = 37), the sum of DAGs with at least 1 SFA (**B**; *n* = 20), and representative species of DAGs containing either SFA (i.e., DAG 32:0; DAG 16:0_16:0; **C**) or omega-3 PUFAs (i.e., DAG 44:12; DAG 22:6_22:6; **D**). Correlation between the sum of all DAG species (*n* = 37) and the sum of DAGs with at least 1 SFA (*n* = 20; **E**). The sum of TAG species with short/medium-chain FAs (**F**; *n* = 9), the sum of TAG species with only SFAs (**G**; *n* = 3), and a representative SFA-containing TAG species TAG 48:0; TAG 16:0_16:0_16:0 (**H**). Lipid intensities are in arbitrary units (A.U.).

Furthermore, we also performed a subanalysis of certain lipid classes with a known relationship to insulin resistance (e.g., diacylglycerols; DAGs) or DNL (e.g., short/medium-chain TAGs containing 38 to 48 carbons and 0 to 3 double bonds; see Section 4 for details) in the liver (Figure 9). We found that krill oil administration reduced the total DAG levels in liver tissue of ω3PL and ω3PL-R mice (Figure 9A), and in particular the content of DAGs containing SFA (Figure 9B,C), while supplementation of omega-3 PUFAs as TAGs in the ω3TG-R group was ineffective. In contrast, ω3TG-R mice showed increased levels of DAG species containing primarily DHA (e.g., DAG 44:12; DAG 22:6_22:6), with lower increases observed in the krill oil-supplemented groups (Figure 9D). However, DAG species containing at least 1 SFA represented the majority of all DAGs (20 out of 37), while the levels of SFA-containing DAGs showed a very strong correlation with the total levels of all DAGs (Figure 9E). In addition, total levels of short-chain TAGs were analyzed and found to be reduced in both krill oil-supplemented groups (i.e., ω3PL, ω3PL-R) compared to LHF-fed controls (Figure 9F), while the strongest effect was observed at the level of only SFA-containing TAGs (Figure 9G,H). No such effects were observed in ω3TG-R mice supplemented with omega-3 PUFAs as TAGs. 

Overall, the above data suggest that krill oil administration has led to profound changes in the levels of complex lipids as well as polar metabolites in the liver, while affecting lipid species that are either involved in the induction of insulin resistance or are established markers of DNL. 

## 4. Discussion

This study aimed to assess the ability of krill oil supplementation to affect NAFLD-related phenotypes in mice with diet-induced obesity and exacerbated NAFLD. At the same time, we wanted to find out whether the effects of krill oil administration on NAFLD are associated with changes in insulin sensitivity, and to look for possible determinants of these effects. Our results clearly demonstrate the ability of krill oil supplementation to alleviate hepatic steatosis, even in a situation when the accumulation of fat in the liver is maximally stimulated due to the combination of high-fat feeding and thermoneutral housing. In contrast, similar doses of omega-3 PUFAs administered via a TAG-based concentrate did not significantly reduce hepatic fat accumulation under the severe obesogenic and steatosis-promoting conditions. In addition, the potent antisteatotic effects of krill oil were observed in a situation when insulin sensitivity in the liver and at the whole-body level was maintained, and which was associated with a hepatic lipidomic signature characterized by reduced concentrations of both short/medium-chain TAGs and total DAGs. 

To evaluate the efficacy of krill oil administration in influencing NAFLD-associated phenotypes, we adopted a recently established model of exacerbated NAFLD in C57BL/6J mice [36], which combines the administration of a lard-based high-fat diet with thermoneutral animal housing (i.e., ambient temperature ~30 °C). However, instead of using the “J” substrain of C57BL/6 mice as in Giles et al. [36], we used the “N” substrain (i.e., C57BL/6N mice) due to its apparent ability to accumulate a larger amount of TAGs in the liver when fed a high-fat diet. In fact, when comparing high-fat diet-fed mice of the C57BL/6J [36] and C57BL/6N [46] substrains, kept at 22 °C, C57BL/6N mice accumulated more fat in the liver despite being fed a corn oil-based diet rich in n-6 PUFA, which has a lower potential to induce hepatic steatosis compared to an SFA-rich lard-based diet [9,10,14]. Indeed, in terms of liver fat accumulation, the use of C57BL/6N mice in the current study led to the induction of very severe hepatic steatosis with TAG levels reaching ~250 mg/g, which was much more than in the corresponding group of C57BL/6J mice in the reference study by Giles et al. (i.e., ~130 mg/g in the HFD group at 30 °C; see Figure 2e in [36]). Thus, the use of the C57BL/6N mouse substrain in combination with thermoneutral housing and lard-based high-fat feeding allowed remarkably high accumulation of TAGs in the liver.

Notwithstanding the above differences in liver fat accumulation between the two substrains of C57BL/6 mice, our current study demonstrated the ability of krill oil administered to C57BL/6N mice to induce strong antisteatotic effects even in the presence of pronounced hepatic steatosis. Although some previous studies have already shown the beneficial effects of dietary omega-3 PUFAs as PLs on liver fat accumulation in various rodent models of obesity [24,25,26,29,30], none of those models reached the level of liver fat accumulation observed in our current study (see above). Even in genetically obese Zucker *fa*/*fa* rats, the fat content in the liver did not exceed 200 mg/g [25], while in mice fed different high-fat diets [24,26,29,30], it ranged from ~50 to ~160 mg/g, depending on the type and percentage of fat in the diet and the duration of its administration. This work, together with previous studies that involved supplementation of omega-3 PUFAs as PLs using either krill oil [24,25,29] or herring meal extract [26,30], thus demonstrates the excellent ability of krill oil to positively affect liver steatosis, at a wide range of tissue concentrations of TAGs. Importantly, in our present study, the antisteatotic effects of krill oil supplementation were observed regardless of whether krill oil was added to the LHF diet from the very beginning of the dietary interventions or after previous administration of the LHF diet when the animals were already obese.

Krill oil, containing significant amounts of PLs, especially phosphatidylcholine [31], was used in the current study to administer primarily EPA and DHA. The type of krill oil used in the current study is characterized by the presence of two main fractions, i.e., phosphatidylcholines and TAGs, which represent 49 and 28%; furthermore, free FAs, DAGs, ether-linked phosphatidylcholines, cholesterol, phosphatidylethanolamines, phosphatidylinositols and lysophosphatidylcholines represent minor fractions of 4.9%, 3.5%, 3.5%, 3.4%, 3.2%, 2.3%, and 2.0%, respectively [31]. Because TAGs, along with ethyl esters, represent lipid classes traditionally used for supplying omega-3 PUFAs into the organism and to treat NAFLD in humans [21,23], we also included in our current study a group of obese mice given EPA and DHA as a TAG-based concentrate. Specifically, we used Epax 3000 TG concentrate with an EPA and DHA content of 29%, which corresponds to the relative content of these FAs in krill oil [47]. Thus, similar amounts of lard in the LHF diet had to be replaced by one or the other concentrate in order to prepare respective supplemented diets. Furthermore, the EPA:DHA ratio was approximately 1.6:1 in both krill oil and Epax 3000 TG, which facilitates the interpretation of our results compared to previous reports (e.g., [26,29]), where EPA and DHA content, as well as their ratio, differed significantly between the omega-3 PUFA concentrates based on TAGs or PLs. However, despite the similarity in the concentration and ratio of EPA and DHA, krill oil and the TAG-based omega-3 PUFA concentrate differed dramatically in terms of their effects on hepatic steatosis. As determined by biochemical analysis of TAG content in the tissue and histological evaluation (i.e., steatosis score), dietary supplementation with krill oil resulted in a 42% reduction in TAG (glycerolipids) accumulation in the liver of obese mice (i.e., ω3PL-R mice), while in mice with omega-3 PUFAs supplemented as TAGs (i.e., ω3TG-R mice) only an insignificant decrease of 5–12% was found. While this difference cannot be explained by the effects on energy intake, body weight or overall adiposity, adipose tissue functionality was improved specifically in krill oil-supplemented mice. A more pronounced reduction in macrophage accumulation in epididymal WAT of ω3PL-R mice was accompanied by higher plasma adiponectin levels (up to ~15 µg/mL) compared to their counterparts treated with the same dose of omega-3 PUFAs given as TAGs. These data are consistent with previous reports documenting the excellent efficacy of krill oil (vs. omega-3 PUFAs as TAGs; [29]) and its dose-dependent effects [24,29] in stimulating plasma adiponectin levels in high-fat diet-fed mice housed under standard thermal conditions. In this context, krill oil administered at approximately 3-fold lower dose to mice fed a 21% fat diet caused an increase in adiponectin levels to only 7.5 µg/mL [24], which is about half of the values achieved in our current study (see above). The role of elevated adiponectin levels in the antisteatotic effects of krill oil supplementation in our study is supported by the presence of a strong negative correlation between plasma adiponectin levels and the degree of liver TAG accumulation. A similar relationship has been shown, for example, in type 2 diabetic patients before and after treatment with insulin sensitizers thiazolidinediones, which resulted in a reduction in liver fat and improved insulin sensitivity while plasma adiponectin increased [48]. Thus, improved WAT function combined with a substantial increase in plasma adiponectin levels may play a role in the potent effects of krill oil supplementation on both liver steatosis and insulin sensitivity in our mice with exacerbated NAFLD.

Adiponectin levels are negatively associated with hepatic and peripheral insulin resistance and hepatic fat content [49,50]. It is known that adiponectin activates AMPK in both skeletal muscle and liver [51]. Activation of AMPK in turn leads to inhibition of acetyl-CoA carboxylase, a key enzyme in the DNL pathway, as well as to induction of FA oxidation and suppression of lipogenic enzymes [51,52,53]. This is in line with our current data and results from previous rodent studies using various forms of omega-3 PUFAs as PLs [29,30,33,34], which show decreased gene expression primarily within the DNL and cholesterol biosynthesis pathways. In addition to effects on FA metabolism pathways, AMPK is also important for the suppressive effect of adiponectin on hepatic glucose production and for maintaining normal fasting glucose levels [54], as well as for the beneficial effect of omega-3 PUFAs on hepatic insulin sensitivity [53]. Our results showing increased plasma adiponectin associated with decreased TAG accumulation and lipogenic gene expression in the liver are therefore consistent with the involvement of the adiponectin-AMPK axis in the antisteatotic effects of krill oil supplementation. Furthermore, activation of this axis could also explain the observed reduction in tissue DAG levels (especially those containing SFAs) in krill oil-supplemented mice. This effect may be directly related to the improvement of hepatic insulin sensitivity, as DAGs are known to be strongly involved in the development of hepatic insulin resistance [53,55]. Because SFA-containing DAGs represented the majority of DAG species in the liver, a marked reduction in total DAG levels in mice supplemented with krill oil may be a direct consequence of its inhibitory effects on DNL (see below).

**Figure 10:**
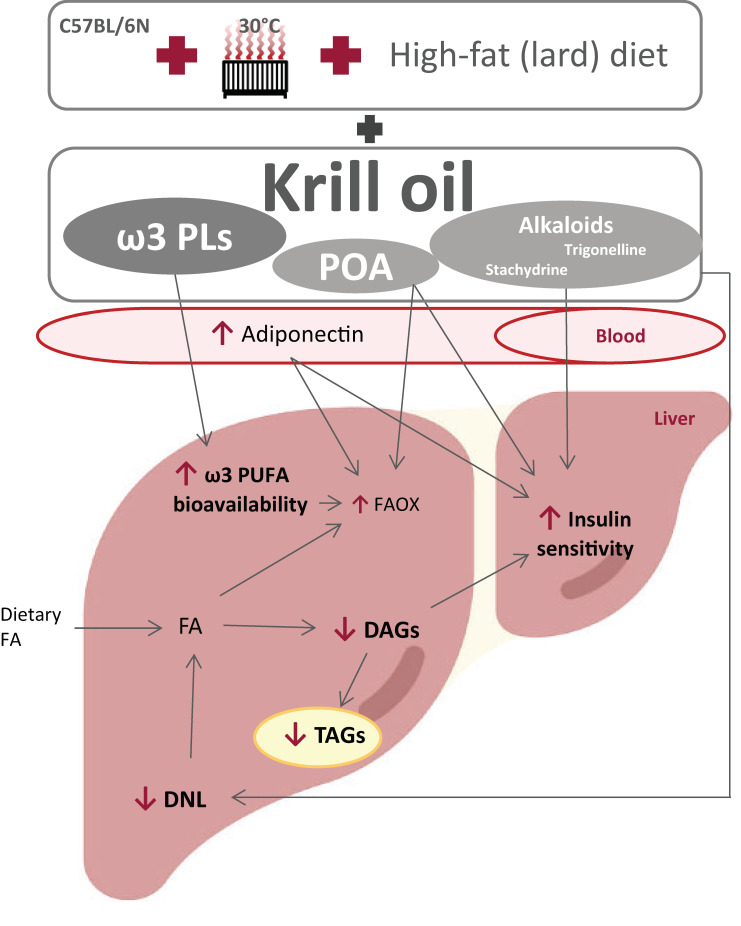
The potential mechanisms involved in the effects of krill oil supplementation on liver fat accumulation and insulin sensitivity in a mouse model of exacerbated hepatic steatosis induced in C57BL/6N mice fed a high-fat (lard) diet in a thermoneutral environment. The effects of krill oil are determined not only by omega-3 PUFA-containing PLs (ω3 PLs) in this marine oil, but also by its other bioactive constituents, including palmitoleic acid (POA) and the alkaloids stachydrine and trigonelline, and may involve direct or indirect mechanisms. The livers of mice fed a high-fat diet supplemented with krill oil have markedly reduced TAG accumulation and improved insulin sensitivity, which is associated with increased bioavailability of omega-3 PUFAs, suppressed DNL, decreased tissue DAG levels, and stimulated FA oxidation (FAOX). While many of these changes may be due to indirect mechanisms based on the beneficial effect of krill oil on WAT functionality associated with markedly elevated plasma adiponectin levels, direct mechanisms may include the effect of stachydrine and/or trigonelline, i.e., alkaloids contained in krill oil, which have previously been shown to have positive effects on NAFLD, presumably by restoring hepatic autophagy.

In addition to elevated adiponectin levels, there are likely to be other mechanisms by which krill oil effectively alleviates exacerbated liver steatosis. Although krill oil supplementation does not seem to change the level of VLDL-TAG secretion (our current data and [34]), it can reduce the mitochondrial citrate carrier activity, as previously observed in rats fed a lard-based high-fat diet [35], and which also showed reduced activities of DNL enzymes such as acetyl-CoA carboxylase and fatty acid synthase. This carrier acts upstream of cytosolic lipogenic processes [56], and its inhibition could thus explain the strong antilipogenic properties of krill oil. In fact, our lipidomics data from the livers of mice fed with krill oil document significantly reduced levels of short/medium-chain TAGs, a subset of TAGs previously proposed as a DNL marker [57]. Therefore, inhibition of this pathway may be one of the main mechanisms of the antisteatotic effects of krill oil. Furthermore, krill oil administration can lead to effective stimulation of FA oxidation in the liver [35], which in turn may be related to the ability of EPA, but not DHA, to increase FA oxidation while inhibiting 1,2-diacylglycerol esterification and thus TAG synthesis in hepatocytes [58]. This is in line with our current and previous [29] results documenting the improved bioavailability of EPA at the level of liver PLs. Furthermore, a recent study on mice fed a high-fat diet based on corn oil suggests that intestinal FA oxidation, which was more effectively stimulated by krill oil compared to omega-3 PUFAs supplemented as TAGs, could also be involved in the antisteatotic effects of this marine oil [59]. It is worth mentioning that other bioactive constituents are present in krill oil, which may possess antisteatotic and insulin-sensitizing properties. Our metabolomics analysis revealed that stachydrine and trigonelline are among the top two polar metabolites that most distinguish the krill oil-supplemented groups from LHF-fed controls, as well as mice supplemented with omega-3 PUFAs as TAGs. Here we show that both of these alkaloids are enriched in the diet supplemented with krill oil, and both have previously been shown to have positive effects on NAFLD, probably by restoring hepatic autophagy [60,61]. The increase in hepatic concentrations of TMAO in mice fed krill oil was probably due to its increased biosynthesis from choline by intestinal bacteria [62]. Moreover, palmitoleic acid contained in krill oil may contribute not only to the positive effects of this marine oil on glucose homeostasis and insulin sensitivity [29], but also on liver steatosis due to its stimulatory effects on PPARα and AMPK activation [63]. The complex composition of krill oil and the role of its constituents in influencing liver fat accumulation and insulin sensitivity is shown in Figure 10 below. Recently, 3-carboxy-4-methyl-5-propyl-2-furanpropanoic acid (CMPF) has been reported as a plasma metabolite whose levels increased with omega-3 PUFAs intake and which was able to alleviate hepatic steatosis when administered to mice [64,65]. Using our metabolomics method we detected CMPF at very low signal intensities in the liver samples. Higher fold changes of 1.7 and 1.9 were observed in the ω3TG-R group compared to the ω3PL and ω3PL-R groups, respectively. However, in the control LHF group, high biological variability of CMPF was noticed, and thus, this metabolite was not ranked among the most discriminating ones.

It is not clear why supplementation with omega-3 PUFAs in the form of a TAG-based concentrate did not reduce liver fat in this model of exacerbated hepatic steatosis, despite the fact that the content of both EPA and DHA in the liver was significantly increased. In this regard, we can speculate that the stronger effects of krill oil on liver steatosis are based on a combination of a number of factors, including higher adiponectin levels along with better bioavailability of EPA in liver tissue, as well as the specific effect of alkaloids contained in krill oil. In addition, choline contained as phosphatidylcholine in krill oil can also contribute to the strong antisteatotic effects of this oil in the liver, as compared to omega-3 PUFAs supplemented as TAGs. Poor availability of hepatic choline/phosphatidylcholine is known to promote steatosis by various mechanisms, including increased DNL and impaired synthesis and secretion of hepatic VLDL (reviewed in [66]). In this context, our previous study in mice fed a corn oil-based high-fat diet showed that the antisteatotic effects of dietary phosphatidylcholine in the liver were unique to PLs containing DHA and EPA, whereas these effects were not present in animals fed soy-derived phosphatidylcholine, which contained mainly PUFAs of n-6 series such as linoleic acid [30]. 

Among the main weaknesses of our study is the fact that, although we used an established model of exacerbated NAFLD, whose characteristics should include NASH and liver fibrosis [36], we were not able to sufficiently induce these characteristics in our experimental mice. While the exact cause is not obvious, it may be related to the fact that a different substrain of C57BL/6 mice was used in our current study. Thus, despite a maximum steatosis score of about 3, LHF-fed control mice of the C57BL/6N substrain showed only minimal lobular inflammation (score < 1), and the overall NAS score of less than 4. This is in sharp contrast to the reference study performed on C57BL/6J mice, where the mean NAS score was ~7 [36]. Therefore, it was not possible to assess the effect of various forms of omega-3 PUFA supplementation on NASH/fibrosis in our current study. Interestingly, however, in LHF-fed control mice, plasma levels of ALT, a marker of liver damage, were almost comparable in our and the reference study. Therefore, the marked decrease in plasma ALT levels in the krill oil-supplemented groups may be due to both the potential protective effect of this oil on liver tissue and its inhibitory effect on hepatic gluconeogenesis [67]. Furthermore, the strengths of our study include: (i) mouse model with marked hepatic steatosis; (ii) evaluation of the relative efficacy of krill oil versus omega-3 TAGs in terms of effects on liver fat; (iii) comprehensive methodological approach, including various in vivo techniques such as hyperinsulinemic-euglycemic clamps, which revealed a number of potential mechanisms of action of krill oil on liver steatosis; and (iv) metabolomic analysis that identified, in addition to omega-3 PLs, other constituents of krill oil that may contribute to the potent antisteatotic effects of this oil.

## 5. Conclusions

By using C57BL/6N mice in combination with thermoneutral housing and lard-based high-fat feeding, we achieved remarkably high levels of TAG accumulation in the liver. Despite these extreme conditions, severe hepatic steatosis was markedly reduced in response to krill oil administration, but not in response to omega-3 PUFAs using a TAG-based concentrate. The potent antisteatotic effects of krill oil, which have been observed in both the prevention and reversal of hepatic steatosis, were associated with improved insulin sensitivity in the liver and at the systemic level. Mechanistically, high plasma adiponectin levels, as well as improved EPA bioavailability, strong repression of DNL, and decreased levels of DAGs in the liver may explain the above beneficial effects of krill oil on liver fat and insulin sensitivity. Furthermore, the role of polar metabolites contained in krill oil, including alkaloids trigonelline and stachydrine, cannot be excluded. Thus, our results suggest that in addition to omega-3 PUFAs contained in PLs, other constituents of krill oil may contribute to its strong antisteatotic effects in the liver.

## Figures and Tables

**Table 1:** Energy balance, adiposity, and biochemical parameters in mice fed high-fat diets supplemented or not with omega-3 PUFA concentrates.

	LHF	ω3PL	ω3PL-R	ω3TG-R
Body weight (g)				
Week 0	23.3 ± 0.7	23.3 ± 0.6	23.1 ± 0.5	23.6 ± 0.7
Week 24	53.6 ± 0.9	48.6 ± 0.9 ^a^	50.5 ± 1.5	53.9 ± 0.8 ^b^
Gain	30.3 ± 0.8	25.3 ± 1.3 ^a^	27.4 ± 1.6 ^b^	30.2 ± 0.4 ^b^
En. intake (MJ/mouse/study)	8.1 ± 0.1	8.1 ± 0.3	7.9 ± 0.3	7.7 ± 0.3
WAT depots (g)				
Epididymal WAT	2.11 ± 0.06	2.54 ± 0.15 ^a^	2.52 ± 0.12 ^a^	2.07 ± 0.07 ^b,c^
Subcutaneous WAT	1.59 ± 0.06	1.45 ± 0.06	1.55 ± 0.06	1.75 ± 0.10
Mesenteric WAT	1.50 ± 0.07	1.09 ± 0.06 ^a^	1.36 ± 0.11 ^b^	1.48 ± 0.07 ^b^
Adiposity index (%)	9.7 ± 0.2	10.5 ± 0.4	10.7 ± 0.4	9.8 ± 0.3
Clinical biochemistry				
TAGs (mmol/L)	1.14 ± 0.12	0.91 ± 0.05	1.02 ± 0.11	0.97 ± 0.04
NEFA (mmol/L)	0.61 ± 0.05	0.57 ± 0.05	0.60 ± 0.07	0.64 ± 0.06
Cholesterol (mmol/L)	6.22 ± 0.18	5.10 ± 0.30 ^a^	5.49 ± 0.14 ^a^	5.87 ± 0.14 ^b^
FBG (mmol/L)	5.19 ± 0.12	4.33 ± 0.09 ^a^	4.46 ± 0.21 ^a^	5.00 ± 0.20 ^b,c^
Insulin (ng/mL)	4.80 ± 0.60	2.65 ± 0.29 ^a^	3.92 ± 0.64	5.74 ± 0.66 ^b^

Data are means ± SEM (*n* = 7–8). Except FBG, measured in overnight fasted mice, biochemical parameters were determined in plasma of mice fed ad libitum. ^a,b,c^ different from LHF, ω3PL, ω3PL-R, respectively (one-way ANOVA or Kruskal–Wallis). FBG, fasting blood glucose; NEFA, non-esterified fatty acids.

## Data Availability

Not applicable.

## Supplementary Materials

The following are available online at https://www.mdpi.com/2072-6643/13/2/437/s1, Table S1: Macronutrient composition of the experimental diets; Table S2: Composition of fatty acids in dietary lipids; Table S3: Gene names and sequences of the oligonucleotide primers; Table S4: Fatty acid composition of the neutral lipid fraction in the liver; Table S5: Fatty acid composition of the polar lipid fraction in the liver; Table S6: Concentration of trigonelline and stachydrine in experimental diets; Table S7: List of annotated complex lipids and polar metabolites in liver samples; Figure S1: Representative histological sections of epididymal white adipose tissue from mice housed in a thermoneutral environment and fed various experimental diets for 24 weeks.
