# Supplementary material for: Krill Oil Supplementation Reduces Exacerbated Hepatic Steatosis Induced by Thermoneutral Housing in Mice with Diet-Induced Obesity
Source: Nutrients. 2021 Jan 29;13(2):437. doi: 10.3390/nu13020437 (PMC7912192; doi:10.3390/nu13020437)
Supplement: Supplementary file 1 [file nutrients-13-00437-s001.pdf]

Supplementary materials

Materials and Methods

Sample extraction for fatty acid composition analysis by gas chromatography

Experimental diet in a powdery form (100 mg; in three aliquots) was mixed with 1.5 mL of NaCl solution (0.73%) and vortexed. After the addition of hexane (5 mL), the sample was sonicated for 10 min and then placed on a shaker for another 2 hours. The mixture was centrifuged at 2,500×*g* for 5 min and then the upper layer transferred to a new (pre-weighed) tube and dried in SpeedVac (35 °C; 2,500 rpm). The residue (the lower layer) was kept in a fume hood for an hour and then mixed with 2 mL of methanol and 4 mL of dichloromethane, vortexed and placed on a shaker for another 30 min. The mix was centrifuged at 2,500×*g* for 5 min and the bottom organic layer then transferred into the tube with the above extract. The sample was dried in SpeedVac (35 °C; 2,500 rpm) and the weight of sample recorded.

Sample extraction for metabolomics and lipidomics analyses of the liver

Liver samples (20 mg) were homogenized with 275 μL methanol containing internal standards (PE 17:0/17:0, PG 17:0/17:0, LPC 17:1, Sphingosine d17:1, Cer d18:1/17:0, SM d18:1/17:0, PC 15:0/18:1-d7, cholesterol-d7, TAG 17:0/17:1/17:0-d_5, DAG 12:0/12:0/0:0, DAG 18:1/2:0/0:0, LPE 17:1, oleic acid-d_9, PI 15:0/18:1-d_7, MAG 17:0/0:0/0:0, PS 17:0/17:0, HexCer d18:1/17:0, DAG 18:1/0:0/18:1-d_5, TAG 20:0/20:1/20:0-d_5, LPG 17:1, LPS 17:1, cardiolipin 16:0/16:0/16:0/16:0) and 275 μL 10% methanol containing internal standards (caffeine-d_9, acetylcholine-d_4, creatinine-d_3, choline-d_9, TMAO-d_9, *N*-methylnicotinamide-d_4, betaine-d_9, butyrobetaine-d_9, creatine-d_3, cotinine-d_3, glucose-d_7, succinic acid-d_4, metformin-d_6) for 1.5 min using a grinder (MM400, Retsch, Germany). Then, 1 mL of MTBE with internal standard (CE 22:1) was added, the tubes were shaken for 1 min and centrifuge at 16,000 rpm for 5 min.

For lipidomic profiling, 100 μL of upper organic phase was collected, evaporated and resuspended using 500 μL methanol with internal standard (CUDA), shaken for 30 s, centrifuged at 16,000 rpm for 5 min and used for LC-MS analysis. An aliquot of 70 μL of bottom aqueous phase was collected, evaporated, resuspended in 70 μL of an acetonitrile/water (4:1, *v*/*v*) mixture with internal standards (CUDA and Val-Tyr-Val), shaken for 30 s, centrifuged at 16,000 rpm for 5 min and analyzed using HILIC metabolomics platform. Another 70 μL of bottom aqueous phase was mixed with 210 μL of an isopropanol/acetonitrile (1:1, *v*/*v*) mixture, shaken for 30 s, centrifuged at 16,000 rpm for 5 min, and the supernatant was evaporated, resuspended in 5% methanol/0.2% formic acid with internal standards (CUDA and Val-Tyr-Val), shaken for 30 s, centrifuged at 16,000 rpm for 5 min and analyzed using HSS T3 metabolomics platform.

LC-MS-based lipidomics

The LC-MS systems consisted of a Vanquish UHPLC System (Thermo Fisher Scientific, Bremen, Germany) coupled to a Q Exactive Plus mass spectrometer (Thermo Fisher Scientific, Bremen, Germany).

Lipids were separated on an Acquity UPLC BEH C18 column (50 × 2.1 mm; 1.7 μm) coupled to an Acquity UPLC BEH C18 VanGuard pre-column (5 × 2.1 mm; 1.7 μm) (Waters, Milford, MA, USA). The column was maintained at 65 °C at a flow-rate of 0.6 mL/min. For LC–ESI(+)-MS analysis, the mobile phase consisted of (A) 60:40 (*v*/*v*) acetonitrile:water with ammonium formate (10 mM) and formic acid (0.1%) and (B) 90:10:0.1 (*v*/*v*/*v*) isopropanol:acetonitrile:water with ammonium formate (10 mM) and formic acid (0.1%). For LC–ESI(−)-MS analysis, the composition of the solvent mixtures were the same with the exception of the addition of ammonium acetate (10 mM) and acetic acid (0.1%) as mobile-phase modifiers. Separation was conducted under the following gradient for LC–ESI(+)-MS: 0 min

15% (B); 0–1 min 30% (B); 1–1.3 min from 30% to 48% (B); 1.3–5.5 min from 48% to 82% (B); 5.5–5.8 min from 82% to 99% (B); 5.8–6 min 99% (B); 6–6.1 min from 99% to 15% (B); 6.1–7.5 min 15% (B). For LC–ESI(−)-MS, the following gradient was used: 0 min 15% (B); 0–1 min 30% (B); 1–1.3 min from 30% to 48% (B); 1.3–4.8 min from 48% to 76% (B); 4.8–4.9 min from 76% to 99% (B); 4.9–5.3 min 99% (B); 5.3–5.4 min from 99% to 15% (B); 5.4–6.8 min 15% (B). A sample volume of 0.5 and 3 μL was used for the injection in ESI(+) and ESI(−), respectively. Sample temperature was maintained at 4 °C.

The ESI source and MS parameters were: sheath gas pressure, 60 arbitrary units; aux gas flow, 25 arbitrary units; sweep gas flow, 2 arbitrary units; capillary temperature, 300 °C; aux gas heater temperature, 370 °C; MS1 mass range, *m/z* 200–1700; MS1 resolving power, 35,000 FWHM (*m/z* 200); number of data-dependent scans per cycle, 3; MS/MS resolving power, 17,500 FWHM (*m/z* 200). For ESI(+), a spray voltage of 3.6 kV and normalized collision energy of 20% was used while for ESI(−) a spray voltage of −3.0 kV and normalized collision energy of 10, 20 and 30% were set-up.

LC-MS-based metabolomics

Polar metabolites were separated on an Acquity UPLC BEH Amide column (50 × 2.1 mm; 1.7 μm) coupled to an Acquity UPLC BEH Amide VanGuard pre-column (5 × 2.1 mm; 1.7 μm) (Waters, Milford, MA, USA). The column was maintained at 45 °C at a flow-rate of 0.4 mL/min. The mobile phase consisted of (A) water with ammonium formate (10 mM) and formic acid (0.125%) and (B) acetonitrile:water (95/5) with ammonium formate (10 mM) and formic acid (0.125%). Separation was conducted under the following gradient: 0 min 100% (B); 0–1 min 100% (B); 1–3.9 min from 100% to 70% (B); 3.9–5.1 min from 70% to 30% (B); 5.1–6.4 min from 30% to 100%(B); 6.4–8.0 min 100% (B). A sample volume of 0.5 μL was used for the injection in ESI(+). Sample temperature was maintained at 4 °C.

Polar metabolites were also separated on an Acquity UPLC HSS T3 column (50 × 2.1 mm; 1.7 μm) coupled to an Acquity UPLC HSS T3 VanGuard pre-column (5 × 2.1 mm; 1.7 μm) (Waters, Milford, MA, USA). The column was maintained at 45 °C using a ramped flow-rate. The mobile phase consisted of (A) water with formic acid (0.2%) and (B) methanol with formic acid (0.1%). Separation was conducted under the following gradient: 0 min 1% (B) 0.3 mL/min; 0–0.5 min 1% (B) 0.3 mL/min; 0.5–2 min from 1% to 60% (B) 0.3 mL/min; 2–2.3 min from 60% to 95% (B) from 0.3 mL/min to 0.5 mL/min; 2.3–3.0 min 95% (B) 0.5 mL/min; 3.0–3.1 min from 95% to 1% (B) 0.5 mL/min; 3.1–4.5 min 1% (B) 0.5 mL/min; 4.5–4.6 min 1% (B) from 0.5 mL/min to 0.3 mL/min; 4.6–5.5 min 1% (B) 0.3 mL/min. A sample volume of 5 μL was used for the injection in ESI(−). Sample temperature was maintained at 4 °C.

The ESI source and MS parameters were: sheath gas pressure, 50 arbitrary units; aux gas flow, 13 arbitrary units; sweep gas flow, 3 arbitrary units; capillary temperature, 260 °C; aux gas heater temperature, 425 °C; MS1 mass range, *m/z* 60–900; MS1 resolving power, 35,000 FWHM (*m/z* 200); number of data-dependent scans per cycle, 3; MS/MS resolving power, 17,500 FWHM (*m/z* 200). A spray voltage of 3.6 kV and −2.5 kV for ESI(+) and ESI(−), respectively, was used. For all metabolomics platforms a normalized collision energy of 20, 30 and 40% was used.

Quality control

Quality control was assured by (i) randomization of the actual samples within the sequence, (ii) injection of quality control (QC) pool samples at the beginning and the end of the sequence and between each 10 actual samples, (iii) analysis of procedure blanks, (iv) serial dilution of QC sample (0, 1/16, 1/8, 1/4, 1/2, 1), (v) checking the peak shape and the intensity of spiked internal standards and the internal standard added prior to injection.

Data processing

LC-MS data from metabolomic and lipidomic profiling were processed through MS-DIAL v. 3.90 software. Metabolites were annotated using in-house retention time–*m/z* library and using MS/MS

libraries available from commercial and open sources (NIST17, MassBank, MoNA). Lipids were annotated using LipidBlast in-built in MS-DIAL [1]. Raw data were filtered using blank samples, serial dilution samples, and QC pool samples with relative standard deviation (RSD) <30%, and then normalized using locally estimated scatterplot smoothing (LOESS) approach by means of QC pool samples injected regularly between 10 actual samples followed by sample-weight normalization. Data were exported as the detector signal intensity in arbitrary units (A.U.).

Quantification of trigonelline and stachydrine in the feed

Feed samples (25 mg) were homogenized with 275 μL methanol and 275 μL 10% methanol for 1.5 min using a grinder. Then, 1 mL of MTBE was added, the tubes were shaken for 1 min and centrifuged at 16,000 rpm for 5 min. Extracts were further processed as for untargeted metabolomics with some modification. Specifically, an aliquot of 70 μL of the bottom aqueous phase was collected, evaporated, resuspended in 150 μL of an acetonitrile/water (4:1, *v*/*v*) mixture with internal standards (CUDA and Val-Tyr-Val), shaken for 30 s, centrifuged at 16,000 rpm for 5 min and analyzed using HILIC metabolomics platform. For quantification, calibration standards were prepared in the feed without trigonelline and stachydrine by spiking the matrix with known concentrations of both analytes. Both trigonelline and stachydrine were detected as protonated molecules (*m*/*z* 138.0549 at RT 3.60 min; *m*/*z* 144.1019 at RT 3.39 min, respectively).

References

[1] H. Tsugawa, T. Cajka, T. Kind, Y. Ma, B. Higgins, K. Ikeda, M. Kanazawa, J. VanderGheynst, O. Fiehn, M. Arita: MS-DIAL: data-independent MS/MS deconvolution for comprehensive metabolome analysis. *Nature Methods* 12 (2015) 523–526; doi: 10.1038/nmeth.3393

Table S1 Macronutrient composition of the experimental diets

	Control	Supplemented diets	
	LHF	ω3PL	ω3TG
Energy density (kJ/g diet)	21.0	19.8	20.2
Dry matter (g/100 g diet)	91.1	89.5	90.0
Macronutrient composition			
Lipids (% of dry matter)	35.4	30.4	32.5
Carbohydrates (% of dry matter)	22.6	25.4	24.8
Proteins (% of dry matter)	20.9	22.2	20.7
Omega-3 concentrates			
Epax 3000 TG (g/100 g diet)*	–	–	11.0
Krill oil (g/100 g diet)†	–	15.0	–
Omega-3 content in the diet			
EPA+DHA (g/100 g diet)#	0.0	3.2	3.2

Omega-3 supplemented diets were based on a commercial high-fat diet (product "DIO - 60 kJ% fat (Lard)"; Cat. No. E15742-34; ssniff Spezialdiäten GmbH, Soest, Germany), in which part of the main lipid component (i.e. lard) has been replaced with a specified amount of Omega-3 concentrate to achieve the same total EPA+DHA content in the respective diets. Information on the content of minerals and vitamins in the LHF diet can be found at: https://www.ssniff.com/documents/03-03%20%20Purified%20DIO%20&%20Controls_v.pdf.

*Product Epax 3000 TG (Epax Norway AS; Ålesund, Norway), containing ~18% EPA and ~11 % DHA.

†Antarctic Krill oil (Rimfrost Sublime; Rimfrost AS; Ålesund, Norway), containing ~13 % EPA and ~8 % DHA.

#Theoretical content of EPA+DHA in the experimental diets, based on the known concentration of EPA and DHA concentration in Omega-3 supplements (as given by the producers).

–, not applicable.

Table S2 Composition of fatty acids in dietary lipids

	Control diet	Omega-3 supplemented diets	
(%)	LHF	ω3PL	ω3TG
SFA			
10:0	0.14 ± 0.00	0.13 ± 0.01	0.12 ± 0.02
12:0	0.15 ± 0.01	0.33 ± 0.02	0.17 ± 0.01
13:0	–	0.02 ± 0.01	0.01 ± 0.00
14:0	1.71 ± 0.1	4.48 ± 0.16	4.14 ± 0.16
15:0	0.05 ± 0.00	0.14 ± 0.01	0.21 ± 0.001
16:0	28.7 ± 0.0	28.1 ± 0.5	26.4 ± 0.6
17:0	0.27 ± 0.01	0.18 ± 0.00	0.30 ± 0.01
17:0, d10 *cis*	0.17 ± 0.00	0.14 ± 0.01	0.19 ± 0.01
18:0	17.3 ± 0.1	12.7 ± 0.1	12.0 ± 0.2
20:0	0.16 ±0.00	0.14 ± 0.01	0.18 ± 0.00
22:0	0.02 ± 0.02	0.07 ± 0.03	0.06 ± 0.00
Total SFA	48.7 ± 0.2	46.4 ± 0.5	43.7 ± 0.7
MUFA			
14:1, d9 *trans*	0.02 ± 0.02	0.05 ± 0.02	0.03 ± 0.00
16:1, d9 *cis*	1.93 ± 0.05	3.44 ± 0.02	4.74 ± 0.01
18:1, oleic	34.4 ± 0.1	29.0 ± 0.3	30.3 ± 0.1
20:1, d11 *cis*	0.44 ± 0.02	0.49 ± 0.03	0.79 ± 0.04
22:1, d13 *cis*	–	0.13 ± 0.01	0.32 ± 0.01
Total MUFA	34.8 ± 0.1	29.7 ± 0.3	31.5 ± 0.2
PUFA (n-6)			
18:2, linoleate	13.1 ± 0.1	13.4 ± 0.2	12.0 ± 0.1
18:3 γ-linolenate	0.04 ± 0.00	0.08 ± 0.01	0.09 ± 0.01
20:2, d11,14 *cis*	0.20 ± 0.01	0.17 ± 0.01	0.20 ± 0.02
20:3, d11,14,17 *cis*	0.06 ± 0.02	0.07 ± 0.001	0.08 ± 0.00
20:3, homo γ-linolenate	0.04 ± 0.00	0.04 ± 0.01	0.06 ± 0.01
20:4, arachidonate	0.08 ± 0.00	0.13 ± 0.01	0.28 ± 0.10
22:4, d7,10,13,16 *cis*	0.03 ± 0.00	0.03 ± 0.01	0.04 ± 0.00

22:5, d4,7,10,13,16 *cis*	–	–	0.05 ± 0.01
Total *n-6* PUFA	13.6 ± 0.2	13.9 ± 0.2	12.8 ± 0.2
PUFA (n-3)			
18:3, α-linolenate	0.96 ± 0.03	1.33 ± 0.03	1.15 ± 0.00
20:5, EPA	–	3.74 ± 0.02	3.93 ± 0.2
22:5, DPA	0.03 ± 0.01	0.07 ± 0.00	0.33 ± 0.03
22:6, DHA	–	1.32 ± 0.04	1.76 ± 0.13
Total *n-3* PUFA	0.99 ± 0.03	6.45 ± 0.05	7.17 ± 0.32

Fatty acid composition (%) in total dietary lipids was determined using gas chromatography (GC). Samples were measured in doublets. Data are means ± SEM.

SFA, saturated fatty acids; MUFA, monounsaturated fatty acids; PUFA, polyunsaturated fatty acids.

–, not detected.

Table S3 Gene names and sequences of the oligonucleotide primers

Name	Abbreviation	5′ sequence	3′ sequence
Acetyl-CoA Carboxylase Alpha	*Acaca*	AGCAGATCCGCAGCTTGGTCC	AGATGGGAGAGGCAGCCCGA
Actin Alpha 2, Smooth Muscle	*Acta2*	AACGAACGCTTCCGCTGCCC	GTGGTTTCGTGGATGCCCGCT
ATP Citrate Lyase	*Acly*	GTGGCGGGGAAGTGCTGTTTGA	TGTGCTCGGGCTGGGAAGGAC
C-C Motif Chemokine Ligand 2	*Ccl2*	CATGCTTCTGGGCCTGCTGTT	CCTGCTGCTGGTGATCCTCTTGTA
CD68 Antigen	*Cd68*	CACTTCGGGCCATGTTTCTCTTG	AGGGGCTGGTAGGTTGATTGTCGT
Collagen Type I Alpha 1 Chain	*Col1a1*	GAAGGGGGCAAAGGTCCCCG	CCGGGAAGACCGACCACACC
Collagen Type III Alpha 1 Chain	*Col3a1*	ATGCCAGCCCCATGACTGTCC	AGGCCCGGCTGGAAAGAAGTC
Elongation Of Very Long Chain Fatty Acids Protein 5	*Elovl5*	CCTCTCGGGTGGCTGTTCTTCC	AGGCTTCGGCTCGGCTTGTC
Farnesyl Diphosphate Synthase	*Fdps*	ATGCCATCAACGACGCTCTGCT	TGGCCCTGGGGTGCTGTCA
Fatty Acid Synthase	*Fasn*	TGGGTGTGGAAGTTCGTCAG	GTCGTGTCAGTAGCCGAGTC
Interleukin 1 Beta	*Il1β*	TCCCCCACACGTTGACAGCTAGG	TCGGCCAAGACAGGTCGCTCA
Secreted Phosphoprotein 1	*Spp1*	ACAGTCGATGTCCCCAACGGC	GGCTGCCCTTTCCGTTGTTGT
Squalene Epoxidase	*Sqle*	GCTTTCTGTATTTTAAACTTGGTGGAGAG	AGTGGAAATAGGATAGAACACGCTTTG
Stearoyl-CoA Desaturase 1	*Scd1*	GCTCCAGCCAAGGTGCCTCTTA	CAGAGTGTCCTCCTGAGCTGATGC
Thrombospondin 1	*Thbs1*	CGAGCACCTGCGGAATGCAC	GCCGATGTGGCGAGGGTCAT
TIMP Metallopeptidase Inhibitor 1	*Timp1*	TTTCCGTTCCTTAGGCGGCCC	GGGTTCCCCAGAAATCAACGAGACC
TIMP Metallopeptidase Inhibitor 2	*Timp2*	AGAGAGCCAAACCGAGCCGTG	TGTGGTGAGGGGTGCTTGGC
TIMP Metallopeptidase Inhibitor 3	*Timp3*	CCTGCCTCACATCAAGGTGCCA	CCTCCTCAACCCAAACAGCCGA

Transforming Growth Factor Beta 1	*Tgfβ1*	GTGGCTGAACCAAGGAGACGGAA	CTCTCCGGTGCCGTGAGCTG
Tumor Necrosis Factor- Alpha	*Tnfα*	AGCTGTCCCCACCTGGCCTCTC	CCCGTGGGGAGCAGAGGTTCAGT

Table S4 Fatty acid composition of the neutral lipid fraction in the liver

	Control	Omega-3 supplemented groups		
(%)	LHF	ω3PL	ω3PL-R	ω3TG-R
SFA				
14:0	0.65 ± 0.03	0.71 ± 0.03	0.72 ±0.03	0.70 ± 0.02
15:0	0.08 ± 0.003	0.12 ± 0.01[a]	0.12 ± 0.01[a]	0.11 ± 0.004[a]
16:0	29.2 ± 0.3	25.8 ± 0.7[a]	25.7 ± 0.6[a]	30.9 ± 0.2[abc]
17:0	0.10 ± 0.01	0.10 ± 0.01	0.09 ± 0.01	0.10 ± 0.00
17:0, d10 *cis*	0.22 ± 0.00	0.26 ± 0.01[a]	0.27 ± 0.02[a]	0.24 ± 0.01[abc]
18:0	1.75 ± 0.06	1.65 ± 0.14	1.63 ± 0.06	1.58 ± 0.04
20:0	0.11 ± 0.01	0.13 ± 0.02	0.12 ± 0.01	0.11 ±0.01
Total SFA	32.1 ± 0.3	28.8 ± 0.7[a]	28.7 ± 0.6[a]	33.7 ± 0.2[abc]
MUFA				
14:1, d9 *trans*	–	–	0.02 ± 0.00	0.02 ± 0.00
16:1, d9 *cis*	6.69 ± 0.33	7.32 ± 0.63	7.24 ± 0.59	7.39 ± 0.22
18:1, oleic	46.9 ± 0.3	43.1 ± 1.2[a]	41.7 ± 1.0[a]	40.7 ± 0.3[a]
20:1, d11 *cis*	0.89 ± 0.02	0.73 ± 0.09[a]	0.59 ± 0.04[ab]	0.65 ± 0.01[ab]
22:1, d13 *cis*	0.03 ± 0.004	0.07 ± 0.01	0.04 ± 0.01	0.03 ± 0.00
Total MUFA	54.5 ± 0.5	51.2 ± 1.8[a]	49.6 ± 1.5[a]	48.8 ± 0.4[a]
PUFA (n-6)				
18:2, linoleate	10.5 ± 0.4	12.2 ± 0.6[a]	12.6 ± 0.8[a]	9.24 ±0.19[abc]
18:3, γ-linolenate	0.19 ± 0.01	0.10 ± 0.01[a]	0.09 ± 0.02[a]	0.07 ± 0.01[a]
20:2, d11,14 cis	0.15 ± 0.02	0.07 ± 0.00[a]	0.06 ± 0.00[a]	0.08 ± 0.01
20:3, homo γ-linolenate	0.40 ± 0.02	0.13 ± 0.02[a]	0.11 ± 0.00[a]	0.18 ± 0.01[abc]
20:4, arachidonate	0.94 ± 0.06	0.24 ± 0.07[a]	0.22 ± 0.02[a]	0.29 ± 0.01[abc]
22:4 d7,10,13,16 *cis*	0.19 ± 0.02	–	0.02 ± 0.00[a]	0.04 ± 0.003[a]
22:5 d4,7,10,13,16 *cis*	0.07 ± 0.01	–	–	0.02 ± 0.003[a]
Total *n-6* PUFA	12.4 ± 0.5	12.7 ± 0.6	13.1 ± 0.8	9.90 ± 0.21[abc]
PUFA (n-3)				
18:3, α-linolenate	0.19 ± 0.01	0.81 ± 0.11[a]	0.91 ± 0.07[a]	0.59 ± 0.03[abc]
20:5, EPA	0.06 ± 0.004	2.09 ± 0.37[a]	2.70 ± 0.26[a]	2.00 ± 0.11[a]

22:5, DPA	0.15 ± 0.02	0.95 ± 0.14[a]	1.12 ± 0.07[a]	1.66 ± 0.07[abc]
22:6, DHA	0.58 ± 0.07	3.33 ± 0.57[a]	3.92 ± 0.44[a]	3.28 ± 0.17[a]
Total *n-3* PUFA	1.01 ± 0.09	7.19 ± 1.15[a]	8.65 ± 0.76[a]	7.52 ± 0.32[a]

Fatty acid composition (%) was determined in the neutral lipid fraction (primarily TAGs) from the liver by gas chromatography (GC).

Data are means ± SEM (*n* = 7-8). SFA, saturated fatty acids; MUFA, monounsaturated fatty acids; PUFA, polyunsaturated fatty acids.

[a,b,c]different from LHF, ω3PL, ω3PL-R, respectively (one-way ANOVA or Kruskal-Wallis test).

–, not detected.

Table S5 Fatty acid composition of the polar lipid fraction in the liver

	Control	Omega-3 supplemented groups		
(%)	LHF	ω3PL	ω3PL-R	ω3TG-R
SFA				
14:0	0.08 ± 0.02	0.08 ± 0.02	0.08 ± 0.01	0.13 ± 0.06
15:0	–	0.06 ± 0.01	0.06 ± 0.02	–
16:0	22.5 ± 0.6	24.4 ± 0.5[a]	24.9 ± 0.6[a]	25.8 ± 0.7[a]
17:0	0.15 ± 0.03	0.19 ± 0.03	0.17 ± 0.00	0.22 ± 0.06
18:0	21.2 ± 0.8	19.5 ± 0.4	19.8 ± 0.6	20.0 ± 0.5
20:0	0.08 ± 0.02	0.14 ± 0.01[a]	0.14 ± 0.01[a]	0.10 ± 0.02
Total SFA	44.0 ± 0.7	44.3 ± 0.6	45.2 ± 0.9	46.3 ± 0.8
MUFA				
16:1	0.24 ± 0.03	0.17 ± 0.02	0.12 ± 0.02	0.17 ± 0.04
16:1, d9 *cis*	0.50 ± 0.07	0.71 ± 0.06	0.65 ± 0.08	0.61 ± 0.04
18:1, d11 *cis*	1.50 ± 0.09	1.51 ± 0.011	1.51 ± 0.15	1.09 ± 0.06[abc]
18:1, d9 *cis*	9.98 ± 0.67	8.73 ± 0.36	8.60 ± 0.31	9.05 ± 0.41
20:1, d11 *cis*	0.15 ± 0.01	0.17 ± 0.03	0.15 ± 0.01	0.16 ± 0.01
Total MUFA	12.4 ± 0.9	11.3 ± 0.5	11.0 ± 0.5	11.1 ± 0.5
PUFA (n-6)				
18:2, linoleate	11.4 ± 0.2	14.4 ± 0.4[a]	14.3 ± 0.6[a]	11.4 ± 0.3[bc]
18:3, γ-linolenate	0.09 ± 0.02	0.06 ± 0.02	–	0.03 ± 0.01
20:2, d11,14, *cis*	0.13 ± 0.01	0.10 ± 0.01	0.11 ± 0.01	0.10 ± 0.02
20:3, d8,11,14 *cis*	1.96 ± 0.07	1.05 ± 0.09[a]	1.05 ± 0.07[a]	1.71 ± 0.06[abc]
20:4, arachidonate	22.0 ± 0.50	5.9 ± 0.2[a]	6.39 ± 0.20[a]	9.43 ± 0.26[abc]
22:4, d7,10,13,16 *cis*	0.26 ± 0.02	–	–	0.04 ± 0.01[a]
22:5, d4,7,10,13,16 *cis*	0.22 ± 0.02	–	–	0.07 ± 0.02[a]
Total *n-6* PUFA	36.0 ± 0.6	21.5 ± 0.5[a]	21.8 ± 0.7[a]	22.8 ± 0.3[a]
PUFA (n-3)				
C18:3, α-linolenate	–	0.06 ± 0.02	0.09±0.02	0.04 ± 0.02
20:5, EPA	0.14 ± 0.01	9.48 ± 0.20[a]	8.86 ± 0.48[a]	6.75 ± 0.14[abc]
22:5, DPA	0.22 ± 0.01	0.88 ± 0.03[a]	0.86 ± 0.05[a]	1.01 ± 0.05[a]

22:6, DHA	7.20 ± 0.23	12.1 ± 0.3[a]	12.2 ± 0.4[a]	12.1 ± 0.5[a]
Total *n-3* PUFA	7.58 ± 0.21	22.9 ± 0.3[a]	22.0 ± 0.6[a]	19.9 ± 0.5[abc]

Fatty acid composition (%) was determined in the polar lipid fraction (primarily PLs) from the liver by gas chromatography (GC).

Data are means ± SEM (n = 7-8). SFA, saturated fatty acids; MUFA, monounsaturated fatty acids; PUFA, polyunsaturated fatty acids.

[a,b,c]different from LHF, ω3PL, ω3PL-R, respectively (one-way ANOVA or Kruskal-Wallis test).

–, not detected.

Table S6 Concentration of trigonelline and stachydrine in experimental diets

	Control	Supplemented diets	
Analyte	LHF	ω3TG	ω3PL
Trigonelline (mg/kg)	<0.02	<0.02	5.6 ± 0.4
Stachydrine (mg/kg)	<0.02	<0.02	1.9 ± 0.2

Data are means ± SEM (n = 3).

Table S7 List of annotated complex lipids and polar metabolites in liver samples

Metabolite name (full)	Class
CAR 2:0; [M]+	CAR
CAR 3:0; [M]+	CAR
CAR 3:0-DC; [M]+	CAR
CAR 4:0; [M]+	CAR
CAR 4:0-OH; [M]+	CAR
CAR 5:0; [M]+	CAR
CAR 5:0-DC; [M]+	CAR
CAR 5:0-OH; [M]+	CAR
CAR 6:0; [M]+	CAR
CAR 6:0-DC; [M]+	CAR
CAR 8:0; [M]+	CAR
CAR 10:0; [M]+	CAR
CAR 14:0; [M]+	CAR
CAR 16:0; [M]+	CAR
CAR 16:1; [M]+	CAR
CAR 18:0; [M]+	CAR
CAR 18:1; [M]+	CAR
CAR 18:2; [M]+	CAR
CAR 20:0; [M]+	CAR
CAR 20:1; [M]+	CAR
CAR 20:5; [M]+	CAR
BMP 40:7; BMP 18:1_22:6; [M+NH4]+	BMP
BMP 42:11; BMP 20:5_22:6; [M+NH4]+	BMP
BMP 44:11; BMP 22:5_22:6; [M+NH4]+	BMP
BMP 44:12; BMP 22:6_22:6; [M+NH4]+	BMP
CE 18:1; [M+NH4]+	CE
CL 68:6; CL 16:1_18:2_16:1_18:2; [M-H]-	CL
CL 70:4; CL 16:0_18:1_18:1_18:2; [M-H]-	CL
CL 70:5; CL 16:1_18:1_18:1_18:2; [M-H]-	CL
CL 70:6; CL 16:1_18:2_18:1_18:2; [M-H]-	CL
CL 70:7; CL 16:1_18:2_18:2_18:2; [M-H]-	CL
CL 72:6; CL 18:1_18:2_18:1_18:2; [M-H]-	CL
CL 72:7; CL 18:1_18:2_18:2_18:2; [M-H]-	CL
CL 72:8; CL 18:2_18:2_18:2_18:2; [M-H]-	CL
CL 74:8; CL 18:1_18:2_18:2_20:3; [M-H]-	CL
CL 74:9; CL 18:2_18:2_18:2_20:3; [M-H]-	CL
CL 76:12; CL 18:2_18:2_18:2_22:6; [M-H]-	CL
Cer-NS d34:1; Cer-NS d18:1/16:0; [M+CH3COO]-	Cer
Cer-NS d36:1; Cer-NS d18:1/18:0; [M+CH3COO]-	Cer
Cer-NS d38:1; Cer-NS d18:1/20:0; [M+CH3COO]-	Cer
Cer-NS d40:1; Cer-NS d18:1/22:0; [M+CH3COO]-	Cer
Cer-NS d40:2; Cer-NS d18:1/22:1; [M+CH3COO]-	Cer
Cer-NS d41:1; Cer-NS d18:1/23:0; [M+CH3COO]-	Cer

Metabolite name (full)	Class
Cer-NS d42:1; Cer-NS d18:1/24:0; [M+CH3COO]-	Cer
Cer-NS d42:2; Cer-NS d18:1/24:1; [M+CH3COO]-	Cer
Cer-NS d42:3; Cer-NS d18:2/24:1; [M+CH3COO]-	Cer
Cholesterol; [M-H2O+H]+	Cholesterol
Cholesterol sulfate; [M-H]-	Cholesterol sulfate
DAG 32:0; DAG 16:0_16:0; [M+NH4]+	DAG
DAG 32:1; DAG 16:0_16:1; [M+NH4]+	DAG
DAG 32:2; DAG 14:0_18:2; [M+NH4]+	DAG
DAG 33:1; DAG 16:0_17:1; [M+NH4]+	DAG
DAG 34:1 (1); DAG 18:0_16:1; [M+NH4]+	DAG
DAG 34:1 (2); DAG 16:0_18:1; [M+NH4]+	DAG
DAG 34:2; DAG 16:0_18:2; [M+NH4]+	DAG
DAG 34:3; DAG 16:1_18:2; [M+NH4]+	DAG
DAG 35:2; DAG 17:1_18:1; [M+NH4]+	DAG
DAG 36:1 (1); DAG 18:0_18:1; [M+NH4]+	DAG
DAG 36:1 (2); DAG 18:0_18:1; [M+NH4]+	DAG
DAG 36:2 (1); DAG 18:0_18:2; [M+NH4]+	DAG
DAG 36:2 (2); DAG 18:1_18:1; [M+NH4]+	DAG
DAG 36:3; DAG 18:1_18:2; [M+NH4]+	DAG
DAG 36:4 (1); DAG 18:2_18:2; [M+NH4]+	DAG
DAG 36:4 (2); DAG 16:0_20:4; [M+NH4]+	DAG
DAG 36:5; DAG 16:0_20:5; [M+NH4]+	DAG
DAG 38:1; DAG 20:0_18:1; [M+NH4]+	DAG
DAG 38:2; DAG 18:1_20:1; [M+NH4]+	DAG
DAG 38:3 (1); DAG 20:1_18:2; [M+NH4]+	DAG
DAG 38:3 (2); DAG 18:0_20:3; [M+NH4]+	DAG
DAG 38:4 (1); DAG 18:1_20:3; [M+NH4]+	DAG
DAG 38:4 (2); DAG 16:0_22:4; [M+NH4]+	DAG
DAG 38:4 (3); DAG 18:0_20:4; [M+NH4]+	DAG
DAG 38:5 (1); DAG 18:1_20:4; [M+NH4]+	DAG
DAG 38:5 (2); DAG 16:0_22:5; [M+NH4]+	DAG
DAG 38:5 (3); DAG 16:0_22:5; [M+NH4]+	DAG
DAG 38:6 (1); DAG 18:1_20:5; [M+NH4]+	DAG
DAG 38:6 (2); DAG 16:0_22:6; [M+NH4]+	DAG
DAG 38:7 (1); DAG 18:2_20:5; [M+NH4]+	DAG
DAG 38:7 (2); DAG 16:1_22:6; [M+NH4]+	DAG
DAG 40:5; DAG 18:1_22:4; [M+NH4]+	DAG
DAG 40:6 (1); DAG 18:1_22:5; [M+NH4]+	DAG
DAG 40:6 (2); DAG 18:0_22:6; [M+NH4]+	DAG
DAG 40:7; DAG 18:1_22:6; [M+NH4]+	DAG
DAG 40:8; DAG 18:2_22:6; [M+NH4]+	DAG
DAG 44:12; DAG 22:6_22:6; [M+NH4]+	DAG
DAGGA 38:6; DAGGA 16:0-22:6; [M-H]-	DAGGA
FA 16:1; [M-H]-	FA
FA 17:1; [M-H]-	FA

Metabolite name (full)	Class
FA 18:1; [M-H]-	FA
FA 18:2; [M-H]-	FA
FA 18:3; [M-H]-	FA
FA 18:4; [M-H]-	FA
FA 19:0; [M-H]-	FA
FA 19:1; [M-H]-	FA
FA 20:0; [M-H]-	FA
FA 20:1; [M-H]-	FA
FA 20:2; [M-H]-	FA
FA 20:3 (1); [M-H]-	FA
FA 20:3 (2); [M-H]-	FA
FA 20:4 (1); [M-H]-	FA
FA 20:4 (2); [M-H]-	FA
FA 20:5 (1); [M-H]-	FA
FA 20:5 (2); [M-H]-	FA
FA 22:1; [M-H]-	FA
FA 22:3; [M-H]-	FA
FA 22:4; [M-H]-	FA
FA 22:5 (1); [M-H]-	FA
FA 22:5 (2); [M-H]-	FA
FA 22:6; [M-H]-	FA
FA 24:1; [M-H]-	FA
FA 24:4; [M-H]-	FA
FA 24:5; [M-H]-	FA
FA 24:6; [M-H]-	FA
HexCer-NS d38:1; [M+CH3COO]-	HexCer
HexCer-NS d40:1; [M+CH3COO]-	HexCer
HexCer-NS d41:1; [M+CH3COO]-	HexCer
HexCer-NS d42:1; [M+CH3COO]-	HexCer
HexCer-NS d42:2; [M+CH3COO]-	HexCer
LPC 16:0; [M+H]+	LPC
LPC 16:1; [M+H]+	LPC
LPC 17:0; [M+H]+	LPC
LPC 18:0; [M+H]+	LPC
LPC 18:1; [M+H]+	LPC
LPC 20:0; [M+H]+	LPC
LPC 20:1; [M+H]+	LPC
LPC 20:3; [M+H]+	LPC
LPC 20:4; [M+H]+	LPC
LPC 20:5; [M+H]+	LPC
LPC 22:5; [M+H]+	LPC
LPC 22:6; [M+H]+	LPC
LPE 16:0; [M+H]+	LPE
LPE 18:0; [M+H]+	LPE
LPE 18:1; [M+H]+	LPE

Metabolite name (full)	Class
LPE 20:4; [M+H]+	LPE
LPE 20:5; [M+H]+	LPE
LPE 22:6; [M+H]+	LPE
LPI 18:0; [M-H]-	LPI
MAG 18:1 (1); [M+Na]+	MAG
MAG 18:1 (2); [M+Na]+	MAG
MAG 18:2; [M+Na]+	MAG
PC 30:0; [M+H]+	PC
PC 32:0; PC 16:0_16:0; [M+H]+	PC
PC 32:1; PC 16:0_16:1; [M+H]+	PC
PC 32:2; [M+H]+	PC
PC 33:1; [M+H]+	PC
PC 33:2; [M+H]+	PC
PC 34:0; [M+H]+	PC
PC 34:1; PC 16:0_18:1; [M+H]+	PC
PC 34:2; PC 16:0_18:2; [M+H]+	PC
PC 34:3 (1); PC 16:1_18:2; [M+H]+	PC
PC 34:3 (2); PC 16:0_18:3; [M+H]+	PC
PC 34:4; [M+H]+	PC
PC 34:5; [M+H]+	PC
PC 35:1; [M+H]+	PC
PC 35:2; [M+H]+	PC
PC 35:3; [M+H]+	PC
PC 35:4; [M+H]+	PC
PC 35:5; [M+H]+	PC
PC 36:1; PC 18:0_18:1; [M+H]+	PC
PC 36:2; PC 18:0_18:2; [M+H]+	PC
PC 36:3 (1); PC 18:1_18:2; [M+H]+	PC
PC 36:3 (2); PC 16:0_20:3; [M+H]+	PC
PC 36:4 (1); PC 18:2_18:2; [M+H]+	PC
PC 36:4 (2); PC 16:0_20:4; [M+H]+	PC
PC 36:4 (3); PC 16:0_20:4; [M+H]+	PC
PC 36:5 (1); [M+H]+	PC
PC 36:5 (2); PC 16:0_20:5; [M+H]+	PC
PC 36:6 (1); [M+H]+	PC
PC 36:6 (2); [M+H]+	PC
PC 37:2; [M+H]+	PC
PC 37:3; [M+H]+	PC
PC 37:4; [M+H]+	PC
PC 37:6 (1); [M+H]+	PC
PC 37:6 (2); [M+H]+	PC
PC 38:1; [M+H]+	PC
PC 38:2; [M+H]+	PC
PC 38:3 (1); PC 18:0_20:3; [M+H]+	PC
PC 38:3 (2); [M+H]+	PC

Metabolite name (full)	Class
PC 38:4 (1); [M+H]+	PC
PC 38:4 (2); PC 18:0_20:4; [M+H]+	PC
PC 38:5 (1); PC 18:1_20:4; [M+H]+	PC
PC 38:5 (2); PC 18:0_20:5; [M+H]+	PC
PC 38:6 (1); PC 18:1_20:5; [M+H]+	PC
PC 38:6 (2); PC 16:0_22:6; [M+H]+	PC
PC 38:7 (1); PC 18:2_20:5; [M+H]+	PC
PC 38:7 (2); PC 16:1_22:6; [M+H]+	PC
PC 38:7 (3); [M+H]+	PC
PC 38:8; [M+H]+	PC
PC 39:4; [M+H]+	PC
PC 39:6; [M+H]+	PC
PC 39:7; [M+H]+	PC
PC 40:3; [M+H]+	PC
PC 40:4 (1); [M+H]+	PC
PC 40:4 (2); [M+H]+	PC
PC 40:5 (1); PC 18:0_22:5; [M+H]+	PC
PC 40:5 (2); [M+H]+	PC
PC 40:6 (1); PC 18:1_22:5; [M+H]+	PC
PC 40:6 (2); [M+H]+	PC
PC 40:6 (3); PC 18:0_22:6; [M+H]+	PC
PC 40:7 (1); [M+H]+	PC
PC 40:7 (2); PC 18:1_22:6; [M+H]+	PC
PC 40:8; PC 20:4_20:4; [M+H]+	PC
PC 40:9; [M+H]+	PC
PC 40:10; [M+H]+	PC
PC 42:6; [M+H]+	PC
PC 42:7; [M+H]+	PC
PC 42:9; [M+H]+	PC
PC 42:10; [M+H]+	PC
PC 42:11; [M+H]+	PC
PC 44:12; [M+H]+	PC
PC 30:0e; [M+H]+	PCe
PC 32:0e; [M+H]+	PCe
PC 32:1e (1); [M+H]+	PCe
PC 32:1e (2); [M+H]+	PCe
PC 34:1e; [M+H]+	PCe
PC 34:2e; [M+H]+	PCe
PC 34:5e; [M+H]+	PCe
PC 36:4e; [M+H]+	PCe
PC 36:5e; [M+H]+	PCe
PC 36:6e (1); [M+H]+	PCe
PC 36:6e (2); [M+H]+	PCe
PC 38:4e; [M+H]+	PCe
PC 38:5e; [M+H]+	PCe

Metabolite name (full)	Class
PC 38:6e (1); [M+H]+	PCe
PC 38:6e (2); [M+H]+	PCe
PC 38:7e; [M+H]+	PCe
PC 40:11e; [M+H]+	PCe
PC 44:12e; [M+H]+	PCe
PE 34:1; PE 16:0_18:1; [M+H]+	PE
PE 34:2; PE 16:0_18:2; [M+H]+	PE
PE 34:3; [M+H]+	PE
PE 36:1; PE 18:0_18:1; [M+H]+	PE
PE 36:2; PE 18:0_18:2; [M+H]+	PE
PE 36:3; PE 18:1_18:2; [M+H]+	PE
PE 36:4; PE 16:0_20:4; [M+H]+	PE
PE 36:5; PE 16:0_20:5; [M+H]+	PE
PE 36:6; [M+H]+	PE
PE 38:3; [M+H]+	PE
PE 38:4 (1); [M+H]+	PE
PE 38:4 (2); PE 18:0_20:4; [M+H]+	PE
PE 38:5 (1); PE 18:1_20:4; [M+H]+	PE
PE 38:5 (2); PE 18:0_20:5; [M+H]+	PE
PE 38:6 (1); PE 18:1_20:5; [M+H]+	PE
PE 38:6 (2); PE 16:0_22:6; [M+H]+	PE
PE 38:7 (1); [M+H]+	PE
PE 38:7 (2); [M+H]+	PE
PE 38:7 (3); [M+H]+	PE
PE 38:8; [M+H]+	PE
PE 38:9; [M+H]+	PE
PE 40:4; [M+H]+	PE
PE 40:5 (1); PE 18:0_22:5; [M+H]+	PE
PE 40:5 (2); [M+H]+	PE
PE 40:6 (1); PE 18:0_22:6; [M+H]+	PE
PE 40:6 (2); [M+H]+	PE
PE 40:7 (1); PE 18:1_22:6; [M+H]+	PE
PE 40:7 (2); [M+H]+	PE
PE 40:8 (1); [M+H]+	PE
PE 40:8 (2); [M+H]+	PE
PE 40:8 (3); [M+H]+	PE
PE 40:9 (1); [M+H]+	PE
PE 40:9 (2); [M+H]+	PE
PE 40:10; [M+H]+	PE
PE 42:9; [M+H]+	PE
PE 42:10; [M+H]+	PE
PE 36:5e; PE 16:1e/20:4; [M-H]-	PEe
PE 36:6e; PE 16:1e/20:5; [M-H]-	PEe
PE 38:5e (1); PE 16:1e/22:4; [M-H]-	PEe
PE 38:5e (2); PE 18:1e/20:4; [M-H]-	PEe

Metabolite name (full)	Class
PE 38:6e; PE 16:1e/22:5; [M-H]-	PEe
PE 38:7e; PE 16:1e/22:6; [M-H]-	PEe
PE 40:5e (1); PE 18:0e/22:5; [M-H]-	PEe
PE 40:5e (2); PE 18:1e/22:4; [M-H]-	PEe
PE 40:5e (3); PE 20:1e/20:4; [M-H]-	PEe
PE 40:6e; PE 18:1e/22:5; [M-H]-	PEe
PE 40:7e; PE 18:1e/22:6; [M-H]-	PEe
PE 40:8e; PE 18:2e/22:6; [M-H]-	PEe
PE 42:7e; PE 20:1e/22:6; [M-H]-	PEe
PG 32:0; PG 16:0_16:0; [M-H]-	PG
PG 32:1; PG 16:0_16:1; [M-H]-	PG
PG 34:1; PG 16:0_18:1; [M-H]-	PG
PG 34:2 (1); PG 16:1_18:1; [M-H]-	PG
PG 34:2 (2); PG 16:0_18:2; [M-H]-	PG
PG 36:2 (1); PG 18:1_18:1; [M-H]-	PG
PG 36:2 (2); PG 18:0_18:2; [M-H]-	PG
PG 36:3 (1); PG 18:1_18:2; [M-H]-	PG
PG 36:3 (2); PG 18:1_18:2; [M-H]-	PG
PG 36:4 (1); PG 18:2_18:2; [M-H]-	PG
PG 36:4 (2); PG 16:0_20:4; [M-H]-	PG
PG 38:4; PG 18:0_20:4; [M-H]-	PG
PG 38:5 (1); PG 18:1_20:4; [M-H]-	PG
PG 38:5 (2); PG 16:0_22:5; [M-H]-	PG
PG 38:6 (1); PG 18:1_20:5; [M-H]-	PG
PG 38:6 (2); PG 16:0_22:6; [M-H]-	PG
PG 38:7; PG 16:1_22:6; [M-H]-	PG
PG 40:5; PG 18:1_22:4; [M-H]-	PG
PG 40:6 (1); PG 18:1_22:5; [M-H]-	PG
PG 40:6 (2); PG 18:1_22:5; [M-H]-	PG
PG 40:7; PG 18:1_22:6; [M-H]-	PG
PG 40:8; PG 18:2_22:6; [M-H]-	PG
PG 42:9; PG 20:3_22:6; [M-H]-	PG
PG 42:10 (1); PG 20:5_22:5; [M-H]-	PG
PG 42:10 (2); PG 20:4_22:6; [M-H]-	PG
PG 42:11; PG 20:5_22:6; [M-H]-	PG
PG 44:11 (1); PG 22:5_22:6; [M-H]-	PG
PG 44:11 (2); PG 22:5_22:6; [M-H]-	PG
PG 44:12; PG 22:6_22:6; [M-H]-	PG
PI 34:2; PI 16:0_18:2; [M-H]-	PI
PI 36:1; PI 18:0_18:1; [M-H]-	PI
PI 36:2; PI 18:0_18:2; [M-H]-	PI
PI 36:3 (1); PI 18:1_18:2; [M-H]-	PI
PI 36:3 (2); PI 16:0_20:3; [M-H]-	PI
PI 36:4; PI 16:0_20:4; [M-H]-	PI
PI 36:5; PI 16:0_20:5; [M-H]-	PI

Metabolite name (full)	Class
PI 37:4; PI 17:0_20:4; [M-H]-	PI
PI 37:5; [M-H]-	PI
PI 38:3 (1); PI 18:0_20:3; [M-H]-	PI
PI 38:3 (2); PI 18:0_20:3; [M-H]-	PI
PI 38:4; PI 18:0_20:4; [M-H]-	PI
PI 38:5 (1); PI 18:1_20:4; [M-H]-	PI
PI 38:5 (2); PI 18:0_20:5; [M-H]-	PI
PI 38:6 (1); PI 18:1_20:5; [M-H]-	PI
PI 38:6 (2); PI 16:0_22:6; [M-H]-	PI
PI 40:4 (1); PI 18:0_22:4; [M-H]-	PI
PI 40:4 (2); PI 20:0_20:4; [M-H]-	PI
PI 40:5 (1); PI 18:0_22:5; [M-H]-	PI
PI 40:5 (2); PI 18:0_22:5; [M-H]-	PI
PI 40:6 (1); PI 18:1_22:5; [M-H]-	PI
PI 40:6 (2); PI 18:0_22:6; [M-H]-	PI
PI 40:7; PI 18:1_22:6; [M-H]-	PI
PMeOH 34:2; PMeOH 16:0_18:2; [M-H]-	PMeOH
PMeOH 36:4; PMeOH 16:0_20:4; [M-H]-	PMeOH
PMeOH 36:5; PMeOH 16:0_20:5; [M-H]-	PMeOH
PS 36:4; PS 16:0_20:4; [M-H]-	PS
PS 36:5; PS 16:0_20:5; [M-H]-	PS
PS 38:4; PS 18:0_20:4; [M-H]-	PS
PS 38:5; PS 18:0_20:5; [M-H]-	PS
PS 38:6; PS 16:0_22:6; [M-H]-	PS
PS 40:6; PS 18:0_22:6; [M-H]-	PS
PS 40:7; [M-H]-	PS
SM d33:1; [M+H]+	SM
SM d34:1; SM d18:1/16:0; [M+H]+	SM
SM d36:1; [M+H]+	SM
SM d38:1; [M+H]+	SM
SM d39:1; [M+H]+	SM
SM d40:1; SM d18:1/22:0; [M+H]+	SM
SM d40:2; SM d18:1/22:1; [M+H]+	SM
SM d41:1; SM d17:0/24:1; [M+H]+	SM
SM d41:2; SM d11:1/30:1; [M+H]+	SM
SM d42:1; SM d18:1/24:0; [M+H]+	SM
SM d42:2; SM d18:1/24:1; [M+H]+	SM
TAG 44:1; TAG 10:0_16:0_18:1; [M+NH4]+	TAG
TAG 46:0; TAG 14:0_16:0_16:0; [M+NH4]+	TAG
TAG 46:1; TAG 14:0_16:0_16:1; [M+NH4]+	TAG
TAG 46:2; TAG 16:0_14:1_16:1; [M+NH4]+	TAG
TAG 46:3; TAG 14:1_16:1_16:1; [M+NH4]+	TAG
TAG 48:0; TAG 16:0_16:0_16:0; [M+NH4]+	TAG
TAG 48:1; TAG 14:0_16:0_18:1; [M+NH4]+	TAG
TAG 48:2; TAG 14:0_16:1_18:1; [M+NH4]+	TAG

Metabolite name (full)	Class
TAG 48:3; TAG 14:0_16:1_18:2; [M+NH4]+	TAG
TAG 48:4; TAG 14:1_16:1_18:2; [M+NH4]+	TAG
TAG 49:1; TAG 15:0_16:0_18:1; [M+NH4]+	TAG
TAG 49:2; TAG 16:0_16:1_17:1; [M+NH4]+	TAG
TAG 49:3; TAG 15:0_16:1_18:2; [M+NH4]+	TAG
TAG 50:0; TAG 16:0_16:0_18:0; [M+NH4]+	TAG
TAG 50:1; TAG 16:0_16:0_18:1; [M+NH4]+	TAG
TAG 50:2; TAG 16:0_16:1_18:1; [M+NH4]+	TAG
TAG 50:3 (1); TAG 16:1_16:1_18:1; [M+NH4]+	TAG
TAG 50:3 (2); TAG 16:0_16:1_18:2; [M+NH4]+	TAG
TAG 50:4 (1); TAG 16:1_16:1_18:2; [M+NH4]+	TAG
TAG 50:4 (2); TAG 16:0_18:1_16:3; [M+NH4]+	TAG
TAG 50:5 (1); TAG 16:1_16:1_18:3; [M+NH4]+	TAG
TAG 50:5 (2); TAG 16:0_18:1_16:4; [M+NH4]+	TAG
TAG 50:6; TAG 16:0_18:2_16:4; [M+NH4]+	TAG
TAG 51:1; TAG 16:0_17:0_18:1; [M+NH4]+	TAG
TAG 51:2; TAG 16:0_17:1_18:1; [M+NH4]+	TAG
TAG 51:3; TAG 16:1_17:1_18:1; [M+NH4]+	TAG
TAG 51:4; TAG 16:1_17:1_18:2; [M+NH4]+	TAG
TAG 52:1; TAG 16:0_18:0_18:1; [M+NH4]+	TAG
TAG 52:2; TAG 16:0_18:1_18:1; [M+NH4]+	TAG
TAG 52:3; TAG 16:0_18:1_18:2; [M+NH4]+	TAG
TAG 52:4 (1); TAG 16:1_18:1_18:2; [M+NH4]+	TAG
TAG 52:4 (2); TAG 16:1_18:1_18:2; [M+NH4]+	TAG
TAG 52:5 (1); TAG 16:1_18:1_18:3; [M+NH4]+	TAG
TAG 52:5 (2); TAG 16:0_16:0_20:5; [M+NH4]+	TAG
TAG 52:6 (1); TAG 16:0_16:1_20:5; [M+NH4]+	TAG
TAG 52:6 (2); TAG 14:0_16:0_22:6; [M+NH4]+	TAG
TAG 52:7; TAG 16:1_18:2_18:4; [M+NH4]+	TAG
TAG 52:8; TAG 18:2_18:2_16:4; [M+NH4]+	TAG
TAG 53:1; TAG 16:0_19:0_18:1; [M+NH4]+	TAG
TAG 53:2; TAG 16:0_18:1_19:1; [M+NH4]+	TAG
TAG 53:3; TAG 17:0_18:1_18:2; [M+NH4]+	TAG
TAG 53:4; TAG 17:1_18:1_18:2; [M+NH4]+	TAG
TAG 53:5; TAG 18:1_17:2_18:2; [M+NH4]+	TAG
TAG 53:6; TAG 16:0_17:1_20:5; [M+NH4]+	TAG
TAG 54:1; TAG 16:0_20:0_18:1; [M+NH4]+	TAG
TAG 54:2; TAG 16:0_18:1_20:1; [M+NH4]+	TAG
TAG 54:3 (1); TAG 18:0_18:1_18:2; [M+NH4]+	TAG
TAG 54:3 (2); TAG 16:0_20:1_18:2; [M+NH4]+	TAG
TAG 54:4 (1); TAG 18:0_18:1_18:3; [M+NH4]+	TAG
TAG 54:4 (2); TAG 18:1_18:1_18:2; [M+NH4]+	TAG
TAG 54:5 (1); TAG 18:0_18:2_18:3; [M+NH4]+	TAG
TAG 54:5 (2); TAG 16:0_18:1_20:4; [M+NH4]+	TAG
TAG 54:5 (3); TAG 16:0_18:1_20:4; [M+NH4]+	TAG

Metabolite name (full)	Class
TAG 54:6 (1); TAG 18:1_18:2_18:3; [M+NH4]+	TAG
TAG 54:6 (2); TAG 16:0_18:1_20:5; [M+NH4]+	TAG
TAG 54:7 (1); TAG 16:1_18:2_20:4; [M+NH4]+	TAG
TAG 54:7 (2); TAG 16:0_18:2_20:5; [M+NH4]+	TAG
TAG 54:8; TAG 16:1_18:2_20:5; [M+NH4]+	TAG
TAG 54:9 (1); TAG 16:1_18:3_20:5; [M+NH4]+	TAG
TAG 54:9 (2); TAG 16:1_18:3_20:5; [M+NH4]+	TAG
TAG 54:10; TAG 16:1_18:4_20:5; [M+NH4]+	TAG
TAG 55:2 (1); TAG 18:0_18:1_19:1; [M+NH4]+	TAG
TAG 55:2 (2); TAG 16:0_18:1_21:1; [M+NH4]+	TAG
TAG 55:3 (1); TAG 18:0_19:1_18:2; [M+NH4]+	TAG
TAG 55:3 (2); TAG 18:1_18:1_19:1; [M+NH4]+	TAG
TAG 55:4; TAG 18:1_19:1_18:2; [M+NH4]+	TAG
TAG 55:6; TAG 16:0_17:1_22:5; [M+NH4]+	TAG
TAG 55:7 (1); TAG 17:1_18:1_20:5; [M+NH4]+	TAG
TAG 55:7 (2); TAG 16:0_17:1_22:6; [M+NH4]+	TAG
TAG 55:8 (1); TAG 17:1_18:2_20:5; [M+NH4]+	TAG
TAG 55:8 (2); TAG 15:0_18:2_22:6; [M+NH4]+	TAG
TAG 56:1 (1); TAG 18:0_20:0_18:1; [M+NH4]+	TAG
TAG 56:1 (2); TAG 16:0_22:0_18:1; [M+NH4]+	TAG
TAG 56:2; TAG 16:0_18:1_22:1; [M+NH4]+	TAG
TAG 56:3; TAG 18:1_18:1_20:1; [M+NH4]+	TAG
TAG 56:4 (1); TAG 18:0_18:2_20:2; [M+NH4]+	TAG
TAG 56:4 (2); TAG 18:1_20:1_18:2; [M+NH4]+	TAG
TAG 56:5; TAG 18:1_18:1_20:3; [M+NH4]+	TAG
TAG 56:6 (1); TAG 18:0_18:1_20:5; [M+NH4]+	TAG
TAG 56:6 (2); TAG 16:0_18:1_22:5; [M+NH4]+	TAG
TAG 56:7 (1); TAG 18:1_18:2_20:4; [M+NH4]+	TAG
TAG 56:7 (2); TAG 16:0_18:2_22:5; [M+NH4]+	TAG
TAG 56:7 (3); TAG 16:0_18:1_22:6; [M+NH4]+	TAG
TAG 56:7 (4); TAG 16:0_18:1_22:6; [M+NH4]+	TAG
TAG 56:8 (1); TAG 16:1_18:2_22:5; [M+NH4]+	TAG
TAG 56:8 (2); TAG 18:1_18:2_20:5; [M+NH4]+	TAG
TAG 56:8 (3); TAG 16:0_18:2_22:6; [M+NH4]+	TAG
TAG 56:9 (1); TAG 18:2_18:2_20:5; [M+NH4]+	TAG
TAG 56:9 (2); TAG 16:1_18:2_22:6; [M+NH4]+	TAG
TAG 56:10 (1); TAG 16:1_18:3_22:6; [M+NH4]+	TAG
TAG 56:10 (2); TAG 16:0_18:4_22:6; [M+NH4]+	TAG
TAG 56:11 (1); TAG 18:2_18:4_20:5; [M+NH4]+	TAG
TAG 56:11 (2); TAG 16:1_18:4_22:6; [M+NH4]+	TAG
TAG 56:11 (3); TAG 14:0_20:5_22:6; [M+NH4]+	TAG
TAG 57:7; TAG 17:1_18:1_22:5; [M+NH4]+	TAG
TAG 57:8; TAG 17:1_18:1_22:6; [M+NH4]+	TAG
TAG 58:1; TAG 18:0_22:0_18:1; [M+NH4]+	TAG
TAG 58:2 (1); TAG 18:0_18:1_22:1; [M+NH4]+	TAG

Metabolite name (full)	Class
TAG 58:2 (2); TAG 16:0_18:1_24:1; [M+NH4]+	TAG
TAG 58:3 (1); TAG 18:0_22:1_18:2; [M+NH4]+	TAG
TAG 58:3 (2); TAG 18:1_18:1_22:1; [M+NH4]+	TAG
TAG 58:4 (1); TAG 18:0_18:1_22:3; [M+NH4]+	TAG
TAG 58:4 (2); TAG 18:1_22:1_18:2; [M+NH4]+	TAG
TAG 58:5 (1); TAG 16:0_18:0_24:5; [M+NH4]+	TAG
TAG 58:5 (2); TAG 18:1_20:1_20:3; [M+NH4]+	TAG
TAG 58:6 (1); TAG 18:0_18:1_22:5; [M+NH4]+	TAG
TAG 58:6 (2); TAG 16:0_18:1_24:5; [M+NH4]+	TAG
TAG 58:6 (3); TAG 18:1_18:1_22:4; [M+NH4]+	TAG
TAG 58:7 (1); TAG 18:0_18:2_22:5; [M+NH4]+	TAG
TAG 58:7 (2); TAG 18:1_18:1_22:5; [M+NH4]+	TAG
TAG 58:7 (3); TAG 18:1_18:1_22:5; [M+NH4]+	TAG
TAG 58:7 (4); TAG 18:0_18:1_22:6; [M+NH4]+	TAG
TAG 58:8 (1); TAG 18:1_18:2_22:5; [M+NH4]+	TAG
TAG 58:8 (2); TAG 18:1_18:2_22:5; [M+NH4]+	TAG
TAG 58:8 (3); TAG 18:1_18:2_22:5; [M+NH4]+	TAG
TAG 58:8 (4); TAG 18:1_18:2_22:5; [M+NH4]+	TAG
TAG 58:9 (1); TAG 18:1_18:3_22:5; [M+NH4]+	TAG
TAG 58:9 (2); TAG 18:1_18:3_22:5; [M+NH4]+	TAG
TAG 58:9 (3); TAG 18:1_18:2_22:6; [M+NH4]+	TAG
TAG 58:10 (1); TAG 18:2_18:3_22:5; [M+NH4]+	TAG
TAG 58:10 (2); TAG 16:0_20:5_22:5; [M+NH4]+	TAG
TAG 58:10 (3); TAG 16:0_20:4_22:6; [M+NH4]+	TAG
TAG 58:11 (1); TAG 18:1_18:4_22:6; [M+NH4]+	TAG
TAG 58:11 (2); TAG 16:0_20:5_22:6; [M+NH4]+	TAG
TAG 58:12 (1); TAG 18:2_18:4_22:6; [M+NH4]+	TAG
TAG 58:12 (2); TAG 16:1_20:5_22:6; [M+NH4]+	TAG
TAG 58:12 (3); TAG 14:0_22:6_22:6; [M+NH4]+	TAG
TAG 58:13; TAG 18:3_18:4_22:6; [M+NH4]+	TAG
TAG 60:6 (1); TAG 18:0_20:1_22:5; [M+NH4]+	TAG
TAG 60:6 (2); TAG 18:1_20:1_22:4; [M+NH4]+	TAG
TAG 60:7 (1); TAG 18:1_20:1_22:5; [M+NH4]+	TAG
TAG 60:7 (2); TAG 18:1_18:1_24:5; [M+NH4]+	TAG
TAG 60:7 (3); TAG 20:0_18:1_22:6; [M+NH4]+	TAG
TAG 60:8 (1); TAG 20:1_18:2_22:5; [M+NH4]+	TAG
TAG 60:8 (2); TAG 18:1_20:1_22:6; [M+NH4]+	TAG
TAG 60:9 (1); TAG 16:0_22:4_22:5; [M+NH4]+	TAG
TAG 60:9 (2); TAG 20:1_18:2_22:6; [M+NH4]+	TAG
TAG 60:10; TAG 16:0_22:5_22:5; [M+NH4]+	TAG
TAG 60:11 (1); TAG 18:2_20:4_22:5; [M+NH4]+	TAG
TAG 60:11 (2); TAG 16:0_22:5_22:6; [M+NH4]+	TAG
TAG 60:12 (1); TAG 18:2_20:4_22:6; [M+NH4]+	TAG
TAG 60:12 (2); TAG 18:1_20:5_22:6; [M+NH4]+	TAG
TAG 60:12 (3); TAG 16:0_22:6_22:6; [M+NH4]+	TAG

Metabolite name (full)	Class
TAG 60:13; TAG 18:2_20:5_22:6; [M+NH4]+	TAG
TAG 60:14; TAG 18:3_20:5_22:6; [M+NH4]+	TAG
TAG 62:7; TAG 18:1_20:1_24:5; [M+NH4]+	TAG
TAG 62:12 (1); TAG 18:2_22:5_22:5; [M+NH4]+	TAG
TAG 62:12 (2); TAG 18:2_22:5_22:5; [M+NH4]+	TAG
TAG 62:12 (3); TAG 18:1_22:5_22:6; [M+NH4]+	TAG
TAG 62:13 (1); TAG 18:2_22:5_22:6; [M+NH4]+	TAG
TAG 62:13 (2); TAG 18:1_22:6_22:6; [M+NH4]+	TAG
TAG 62:14; TAG 18:2_22:6_22:6; [M+NH4]+	TAG
TAG 62:15; TAG 18:3_22:6_22:6; [M+NH4]+	TAG
TAG 62:16; TAG 20:5_20:5_22:6; [M+NH4]+	TAG
TAG 68:2; TAG 16:0_18:1_34:1; [M+NH4]+	TAG
TAG 70:3; TAG 18:1_18:1_34:1; [M+NH4]+	TAG
TAG 52:2e; TAG 18:1e_16:0_18:1; [M+NH4]+	TAGe
1-Methyladenosine; [M+H]+	Polar
1-Methylhistamine; [M+H]+	Polar
1-Methylhistidine; [M+H]+	Polar
1-Methylnicotinamide; [M+H]+	Polar
2-Aminoadipic acid; [M+H]+	Polar
2-Hydroxybutyric acid; [M-H]-	Polar
2-Phenylbutyric acid; [M-H]-	Polar
3'-Dephosphocoenzyme A; [M-H]-	Polar
3-Hydroxybutyric acid; [M-H]-	Polar
3-Hydroxyisobutyric acid; [M-H]-	Polar
3-Indoxylsulfate; [M-H]-	Polar
3-Methylhistidine; [M+H]+	Polar
3-Ureidopropionic acid; [M+H]+	Polar
4-Guanidinobutyric acid; [M+H]+	Polar
4-Hydroxyphenyllactic acid; [M-H]-	Polar
5'-S-methyl-5'-thioadenosine; [M+H]+	Polar
7,8-Dihydrobiopterin; [M+H]+	Polar
Adenine; [M+H]+	Polar
Adenosine; [M+H]+	Polar
Adenosine 3'-phosphate 5'-phosphosulfate; [M-H-SO3]-	Polar
Adenosine 5'-diphosphoribose; [M+H]+	Polar
AMP; [M+H]+	Polar
Adenosine 5'-phosphosulfate; [M+OH]-	Polar
Adenylosuccinic acid; [M-H]-	Polar
Ala-Gln; [M+H]+	Polar
Ala-Lys; [M+H]+	Polar
Alanine; [M+H]+	Polar
Arg-Ala; [M+H]+	Polar
Asparagine; [M+H]+	Polar
Aspartic acid; [M-H]-	Polar
Azelaic acid; [M-H]-	Polar

Metabolite name (full)	Class
Betaine; [M]+	Polar
Carnitine; [M+H]+	Polar
Choline; [M]+	Polar
Citric acid; [M-H]-	Polar
Citrulline; [M+H]+	Polar
CMPF; [M-H]-	Polar
Creatine; [M+H]+	Polar
Creatinine; [M+H]+	Polar
Cytidine 5'-diphosphocholine; [M+H]+	Polar
Cytidine 5'-monophosphate; [M-H]-	Polar
Cytosine; [M+H]+	Polar
Dihydrouracil; [M+H]+	Polar
Dimethylarginine; [M+H]+	Polar
Disaccharide (1); [M+NH4]+	Polar
Disaccharide (2); [M+NH4]+	Polar
Ethyl sulfate; [M-H]-	Polar
Ethyl-beta-glucuronide; [M-H]-	Polar
Fumaric acid; [M-H]-	Polar
Gln-Lys; [M+H]+	Polar
Glu-Leu; [M-H]-	Polar
Glu-Val; [M-H]-	Polar
Glucose; [M+Na]+	Polar
Glucuronic acid; [M-H]-	Polar
Glutamic acid; [M+H]+	Polar
Glutamine; [M+H]+	Polar
Glutathione (oxidized); [M+H]+	Polar
Glutathione (reduced); [M+H]+	Polar
Gly-Arg; [M+H]+	Polar
Gly-Met; [M-H]-	Polar
Glycero-3-phosphocholine; [M+H]+	Polar
Glycine; [M+H]+	Polar
Guanine; [M+H]+	Polar
Guanosine; [M+H]+	Polar
Guanosine 5'-monophosphate; [M+H]+	Polar
Hexanoylglycine; [M-H]-	Polar
Hippuric acid; [M-H]-	Polar
Histidine; [M+H]+	Polar
Hypoxanthine; [M+H]+	Polar
Ile-Arg; [M+H]+	Polar
Ile-Ile; [M+H]+	Polar
Inosine; [M-H]-	Polar
Inosine 5'-monophosphate; [M+H]+	Polar
Isoleucine; [M+H]+	Polar
Itaconic acid; [M-H]-	Polar
Lactic acid; [M-H]-	Polar

Metabolite name (full)	Class
Lactobionic acid; [M-H]-	Polar
Leucine; [M+H]+	Polar
Lysine; [M+H]+	Polar
Malic acid; [M-H]-	Polar
Methionine; [M+H]+	Polar
N1-Acetylspermidine; [M+H]+	Polar
N6,N6,N6-Trimethyllysine; [M+H]+	Polar
N6-(1-Iminoethyl)lysine; [M+H]+	Polar
N,N-Dimethylglycine; [M+H]+	Polar
N-(4-Aminobenzoyl)glutamic acid; [M-H]-	Polar
N-Acetylaspartic acid; [M-H]-	Polar
N-Acetylglutamic acid; [M-H]-	Polar
N-Acetylglutamine; [M+Na]+	Polar
N-Acetylhistidine; [M+H]+	Polar
N-Acetyllactosamine; [M+Na]+	Polar
N-Acetylmethionine; [M-H]-	Polar
N-Acetylornithine; [M+H]+	Polar
N-Acetylphenylalanine; [M-H]-	Polar
N-Cinnamoylglycine; [M-H]-	Polar
N-Glycolylneuraminic acid; [M-H]-	Polar
N-Isobutyrylglycine; [M-H]-	Polar
N-Isovalerylglycine; [M-H]-	Polar
N-Methylhistidine; [M+H]+	Polar
N-Tigloylglycine; [M-H]-	Polar
N-alpha-Acetylarginine; [M+H]+	Polar
N-alpha-Acetyllysine; [M+H]+	Polar
N-epsilon-Methyllysine; [M+H]+	Polar
Nicotinamide; [M+H]+	Polar
Nicotinamide riboside cation; [Cat]+	Polar
Ophthalmic acid; [M+H]+	Polar
Ornithine; [M+H]+	Polar
Pantothenic acid; [M+H]+	Polar
Phenaceturic acid; [M-H]-	Polar
Phenylalanine; [M+H]+	Polar
Pimelic acid; [M-H]-	Polar
Pipecolic acid; [M+H]+	Polar
Proline; [M+H]+	Polar
Pyruvic acid; [M-H]-	Polar
Pentose X-phosphate; [M-H]-	Polar
S-Adenosylhomocysteine; [M+H]+	Polar
S-Adenosylmethionine; [M+H]+	Polar
Saccharopine; [M+H]+	Polar
Sebacic acid; [M-H]-	Polar
Ser-Leu; [M-H]-	Polar
Serine; [M+H]+	Polar

Metabolite name (full)	Class
Stachydrine (proline betaine); [M+H]+	Polar
Succinic acid; [M-H]-	Polar
TMAO; [M+H]+	Polar
Taurine; [M+H]+	Polar
Taurocholic acid; [M+H]+	Polar
Tauromuricholic acid (isomer 1); [M+H]+	Polar
Tauromuricholic acid (isomer 2); [M+H]+	Polar
Tauroursodeoxycholic acid; [M+H]+	Polar
Tetrasaccharide; [M+HCO2]-	Polar
Thiamine; [M]+	Polar
Thr-His; [M+H]+	Polar
Thr-Tyr; [M-H]-	Polar
Threonic acid; [M-H]-	Polar
Threonine; [M+H]+	Polar
Trigonelline; [M+H]+	Polar
Trisaccharide; [M+NH4]+	Polar
Tryptophan; [M+H]+	Polar
Tyrosine; [M+H]+	Polar
Uracil; [M+H]+	Polar
Urea; [M+H]+	Polar
Uric acid; [M-H]-	Polar
Uridine; [M-H]-	Polar
Uridine 5'-monophosphate; [M+H]+	Polar
Uridine 5'-diphosphoacetylglucosamine; [M+H]+	Polar
Val-Arg; [M+H]+	Polar
Val-Leu; [M-H]-	Polar
Val-Phe; [M-H]-	Polar
Valine; [M+H]+	Polar
Xanthine; [M-H]-	Polar
Xanthosine; [M+H]+	Polar
alpha-Hydroxyglutaric acid; [M-H]-	Polar
alpha-Ketoglutaric acid; [M-H]-	Polar
NAD; [M+H]+	Polar
NADH; [M-H]-	Polar
gamma-Butyrobetaine; [M]+	Polar
gamma-Glutamylleucine; [M-H]-	Polar
trans-Crotonobetaine; [M]+	Polar

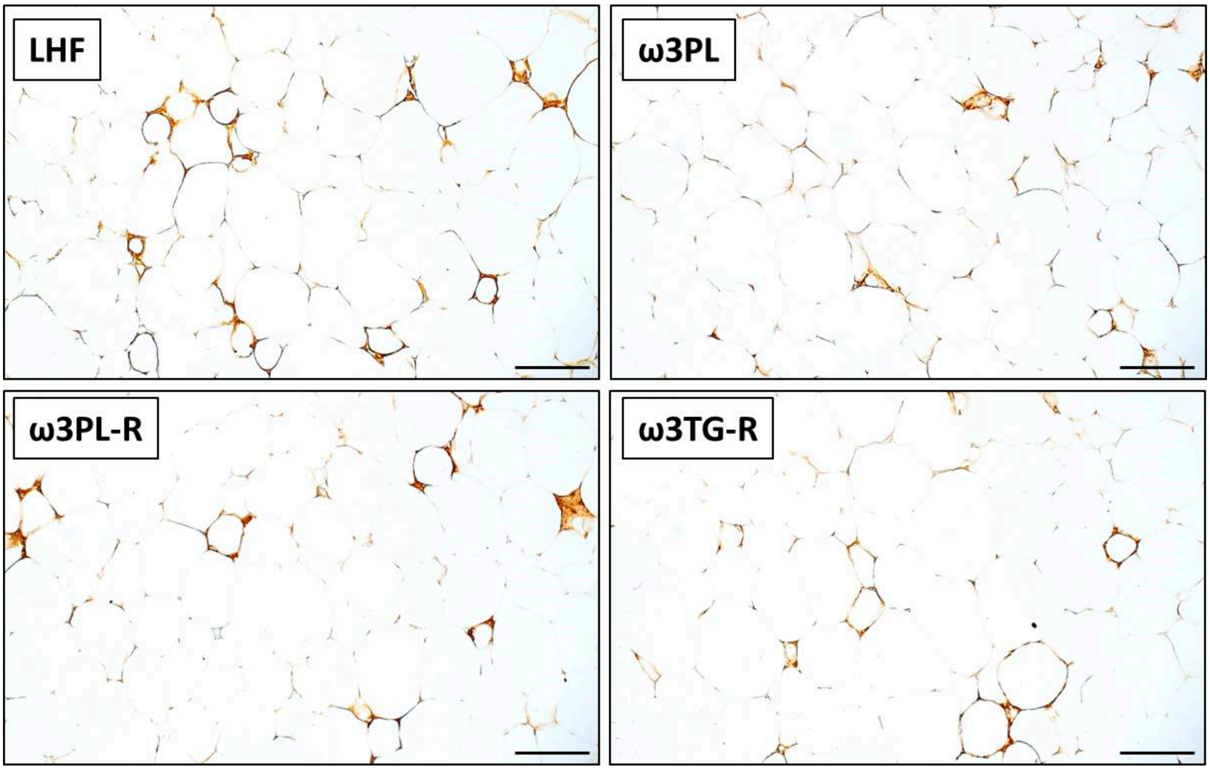

Supplementary Figure 1. Representative histological sections of epididymal white adipose tissue from mice housed in a thermoneutral environment and fed various experimental diets for 24 weeks. Sections were stained with antibody against macrophage marker MAC-2/galectin-3. Bars = 100 μm.

LHF, control mice fed a lard-based high-fat (i.e. LHF) diet; ω3PL, mice fed a LHF-based diet supplemented with Omega-3 PLs in the form of krill oil (i.e. ω3PL diet) for the duration of the experiment (i.e. "preventive" approach); ω3PL-R, mice fed the LHF diet for the first 8 weeks and then from the 9th week the ω3PL diet until the end of the experiment (i.e. "reverse" approach; marked with the letter "R" at the end of the group name); ω3TG-R, mice fed the LHF diet for the first 8 weeks and then from the 9th week the LHF-based diet supplemented with Omega-3 in the form of a concentrate of re-esterified TAGs (i.e. ω3TG diet) until the end of the experiment.
